# Snailase: A Promising Tool for the Enzymatic Hydrolysis of Flavonoid Glycosides From Plant Extracts

**DOI:** 10.3389/fpls.2022.889184

**Published:** 2022-06-09

**Authors:** Christoph Kornpointner, Jakob Scheibelreiter, Heidi Halbwirth

**Affiliations:** Institute of Chemical, Environmental and Bioscience Engineering, Technische Universität Wien, Vienna, Austria

**Keywords:** snailase, enzymatic hydrolysis, flavonoid aglycones, acidic hydrolysis, flavonoids, anthochlors, β-glucosidase, dihydrochalcone

## Abstract

Plants typically contain a broad spectrum of flavonoids in varying concentrations. As a rule, several flavonoid classes occur in parallel, and, even for a single flavonoid, divergent glycosylation patterns are frequently observed, many of which are not commercially available. This can be challenging in studies in which the distribution between flavonoid classes, or features that are not affected by glycosylation patterns, are adressed. In addition, hydrolysis simplifies the quantification process by reducing peak interferences and improving the peak intensity due to the accumulation of the respective aglycone. Effective removal of glycose moieties can also be relevant for technological applications of flavonoid aglycones. Herein, we present a fast and reliable method for the enzymatic hydrolysis glycosides from plant extracts using the commercial enzyme mix snailase, which provided the highest aglycone yields across all investigated flavonoids (aurones: leptosidin, maritimetin, sulfuretin; chalcones: butein, lanceoletin, okanin, phloretin; dihydroflavonols: dihydrokaempferol; flavanones: eriodictyol, hesperetin; flavones: acacetin, apigenin, diosmetin, luteolin; flavonols: isorhamnetin, kaempferol, myricetin, quercetin; isoflavones: biochanin A, formononetin, genistein) from methanolic extracts of nine plants (*Bidens ferulifolia, Coreopsis grandiflora, Fagus sylvatica, Malus* × *domestica, Mentha* × *piperita, Petunia* × *hybrida, Quercus robur, Robinia pseudoacacia*, and *Trifolium pratense*) in comparison to four other enzymes (cellobiase, cellulase, β-glucosidase, and pectinase), as well as to acidic hydrolysis by hydrochloric acid.

## Introduction

Flavonoids are one of the largest groups of secondary metabolites in higher plants, with more than 8,000 individual compounds described to date (Jucá et al., 2020). For a better overview, flavonoids are grouped into different classes according to the oxidation status and substitution pattern of their heterocyclic ring (ring C), e.g., anthocyanidins, flavonols, flavones, isoflavones, dihydroflavonols, or flavanones, and the flavonoid relatives, aurones, and chalcones. Flavonoids are typically found as *O*-glycosides or *C*-glycosides in flowers, leaves, or fruits (Pietta, 2000; Veitch and Grayer, 2011) and fulfill a great variety of important functions in plants. To name only a few, they function as pigments and are responsible for the flavor in flowers and fruits to attract pollinators and seed dispersers (Griesbach, 2005), protect the plants against UV radiation (Takahashi et al., 1991) or freezing (Samanta et al., 2011), and act as detoxifying agents, phytoalexins, or signal molecules (Panche et al., 2016). In addition, the flavonoids present in plant-derived food contribute to human health and have several positive effects. For instance, they are well known for their antioxidant potential, as described in several studies, and their antibacterial, antifungal, anti-inflammatory, antimicrobial, or antiviral activities, as well as cardioprotective effects (Jucá et al., 2020).

From literature, it is obvious that many trees or flowers can accumulate numerous glycosides of a single flavonoid, e.g., acacetin glycosides in *Robinia pseudoacacia* leaves (Veitch et al., 2010), myricetin glycosides in *Carpinus betulus* L. leaves (Hofmann et al., 2016), cyanidin glycosides in *Alstroemeria* flowers (Saito et al., 1985), kaempferol glycosides in *Fagus sylvatica* leaves (Formato et al., 2021) and quercetin glycosides in *Rudbeckia hirta* petals (Schlangen et al., 2009) or *Quercus ilex* L. leaves (Brossa et al., 2009). Due to this variety and high number of accumulated glycosides, the quantification of flavonoids in plant extracts is challenging and the method development can be time and resource consuming. In addition, for the majority of flavonoid glycosides, reference compounds for identification are not commercially available.

To simplify the quantification process, flavonoid glycosides are most commonly hydrolyzed to their respective aglycones. This not only reduces peak interferences during analysis but can also improve the overall concentration by accumulating the respective aglycone. Apart from that, removing the sugar moiety of the flavonoid glycoside has further advantages, for instance, it is reported to increase the antioxidant capacity (de Araújo et al., 2013).

The hydrolysis can be carried out chemically by strong inorganic acids with a concentration of 1–2 M (Hertog et al., 1992; Nuutila et al., 2002; Sainz Martinez et al., 2021). However, different types of flavonoids require different hydrolysis conditions (Hertog et al., 1992; Ahn-Jarvis et al., 2019), and additionally, the rate of acidic hydrolysis depends on the type of glycoside residue, as well as on the binding site on the individual flavonoid. For instance, the type of flavonoid is of importance as flavonol glycosides are hydrolyzed faster than anthocyanins due to the stabilizing effects of the flavylium cation at lower pH (Harborne, 1965). Further, it is also described to rely on the investigated plant material and the hydrolysis should be optimized separately for every plant raw material (Nuutila et al., 2002). Hence, a standardized protocol for acidic hydrolysis of flavonoid glycosides is not available and is not feasible for phytochemical studies that focus on various flavonoid types within even one plant type, let alone more types.

In contrast, sugar moieties can be removed enzymatically with hydrolases. Several enzymes seem to be suitable for flavonoid hydrolysis from natural sources. Enzymatic hydrolysis with pectinase and cellulase of flavonoid glycosides from bergamot peel extracts have been reported (Mandalari et al., 2006). Both enzymes were additionally described to hydrolyze flavonol glycosides from cactus peer fruits and onions (Moussa-Ayoub et al., 2011). Furthermore, β-glucosidase was described for the deglycosylation of flavonoids from extracts of Sasamayu cocoon shells (Kurioka and Yamazaki, 2002) and willow bark (Freischmidt et al., 2015). In addition, naringinase, a mixture of α-l-rhamnosidase and β-d-glucosidase, was previously used for the hydrolysis of flavanol and flavonol glycosides from petunia flower extracts (Haselmair-Gosch et al., 2018); however, the mix is currently not commercially feasible.

Recently, the enzyme mix, snailase, was used to enzymatically hydrolyze purified flavonoid glycosides, for instance, epimedium flavonoids (Jin et al., 2013; Liu et al., 2019), isoflavonoids (Wang et al., 2019), or rutin (quercetin-3-*O*-rutinoside) to its aglycone quercetin (Wang et al., 2011). Snailase is a complex mixture of more than 20 enzymes, including cellulase, invertase, hemicellulase, pectinase, polygalacturonase, protease, among others (You et al., 2011; Jiugang et al., 2012), which is obtained by extraction from the digestive tracts or the crop of mollusks from the genus *Limax* (Jiugang et al., 2012; Wang et al., 2013). However, snailase has not been utilized for the hydrolysis of flavonoid glycosides from raw alcoholic plant extracts before now.

**Figure 1:**
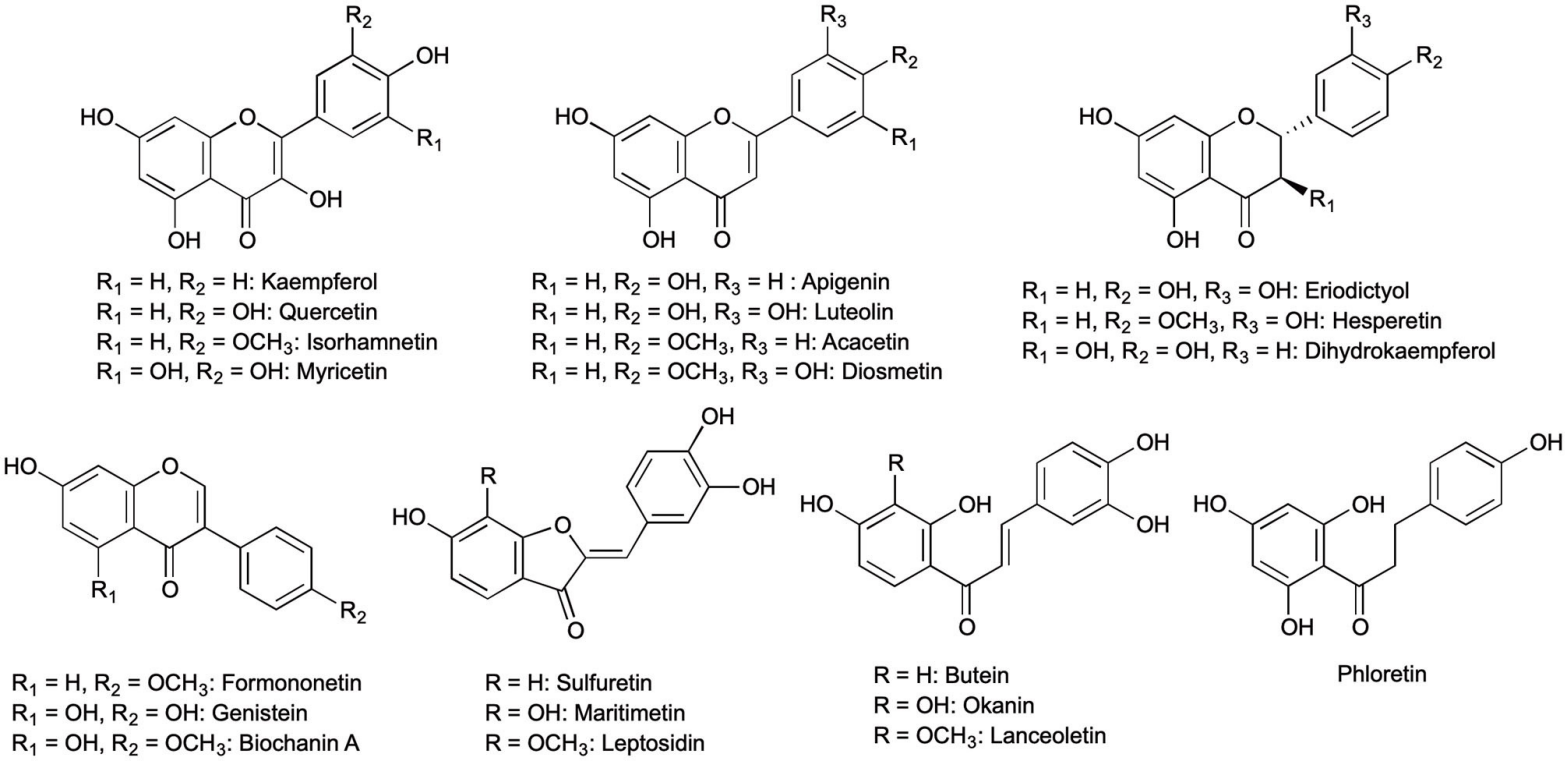
All 21 flavonoid aglycones found in methanolic extracts of *Bidens ferulifolia, Coreopsis grandiflora, Fagus sylvatica, Malus* × *domestica, Mentha* × *piperita, Petunia* × *hybrida, Quercus robur, Robinia pseudoacacia*, and *Trifolium pratense*.

The aim of this study was to develop a fast protocol for the hydrolysis of flavonoid glycosides from plant extracts, which can be employed for different types of flavonoids and raw materials for comparative studies. Therefore, the optimization and efficiency comparison of flavonoid deglycosylation by enzymatic hydrolysis with snailase and four other enzymes (cellobiase, cellulase, β-glucosidase, pectinase), as well as acidic hydrolysis with hydrochloric acid as a reference, were carried out. The yields of various flavonoid aglycones (Figure 1) from four flower extracts (*Bidens ferulifolia, Coreopsis grandiflora, Petunia* × *hybrida, Trifolium pratense*) and five leaf extracts (*Fagus sylvatica, Malus* × *domestica, Mentha* × *piperita, Quercus robur, Robinia pseudoacacia*) were investigated. In addition, various parameters during enzymatic and acidic hydrolysis, e.g., temperature, time, and pH during hydrolysis, as well as concentration of enzyme or acid were varied and tested during this study.

## Materials and Methods

### Plant Material and Extraction

The plant material was collected between 2017 and 2021, shock-frozen with liquid nitrogen, and stored at −80°C until use. Flower petals of *B. ferulifolia* cv. Giant*, C. grandiflora* cv. Early Sunrise, and *P*. × *hybrida* cv. Viva Orange, were collected from our experimental field in Vienna and leaves of *M*. × *domestica* from the experimental orchard of the University of Natural Resources and Life Sciences. Flowers of *T. pratense* were collected in public places in Vienna. Leaves of *F. sylvatica, Q. robur*, and *R. pseudoacacia* were collected in green areas of Gerasdorf (Austria). A pot of *Mentha* × *piperita* was purchased from OBI Baumarkt in 1100 Vienna.

The frozen plant raw material was ground to a fine powder using a mortar and pestle. Subsequently, 0.25–0.35 g of homogenized frozen plant material were extracted with 3.0 ml of methanol (analytical grade, Merck, Germany) at room temperature under magnetic stirring (1,000 rpm) for 2 h, filtered through a 0.22 μm filter, and stored at −20°C until use. All extractions were carried out in triplicates.

### Enzymatic and Acidic Hydrolysis

For enzymatic hydrolysis, glucosidase (cellobiase) from *Aspergillus niger* (≥750 U/g, Sigma-Aldrich, USA), cellulase from *Trichoderma reesei* (≥700 U/g, δ = 1.10–1.30 g/ml, Sigma-Aldrich, USA), β-glucosidase from almonds (≥4 U/mg, Sigma-Aldrich, USA), pectinase from *Aspergillus aculeatus* (≥3,800 U/ml, Sigma-Aldrich, USA), and snailase (Abbexa, UK) were purchased.

Enzymatic hydrolysis was carried out as previously reported (Haselmair-Gosch et al., 2018) with some modifications. Briefly, 20 μl of methanolic extracts were mixed with 20 U (100 U/ml) of cellulase, 20 U pectinase, 20 U β-glucosidase, or 2–5 mg of snailase, respectively, and McIlvaine buffer (0.1 M with 20 μM of sodium ascorbate; ≥99%, Roth, Germany) was added to reach a final volume of 200 μl. In addition, hydrolysis by 5 U cellobiase (50 U/ml) was performed with 10 μl sample and 100 μl total volume due to the lower initial enzyme concentration. Temperature, time, and pH during enzymatic hydrolysis for plant extracts were varied for snailase (Supplementary Tables S1, S2), cellobiase (Supplementary Tables S3, S4), cellulase (Supplementary Tables S5, S6), β-glucosidase (Supplementary Tables S7, S8), and pectinase (Supplementary Tables S9, S10). The hydrolysis was stopped by addition of 30 μl of methanol. The assay was centrifuged for 5 min at 14,000 rpm and 200 μl of the supernatant were mixed with 100 μl of acetonitrile (0.1% formic acid). The initial parameters for enzymatic hydrolysis were based on the supplier information (Abbexa, 2022; Sigma-Aldrich, 2022a,b,c,d).

Acidic hydrolysis was modified according to literature (Hertog et al., 1992; Moussa-Ayoub et al., 2011; Sainz Martinez et al., 2021). In total, 50 μl of plant extract were mixed with 200 μl of 1–2 M HCl (Merck, Germany) at 40, 70, and 100°C for 60 or 120 min (Supplementary Tables S11, S12).

### Quantification by HPLC Analysis

The high-performance liquid chromatography (HPLC) analysis of flavonoid aglycones was carried out on a Dionex UltiMate^©^ RSLC System connected to a DAD-3000RS Photodiode Array Detector (Thermo Scientific, Germany). A Dionex Acclaim^™^ RSLC 120 C18 (2.2 μm, 120 Å, 2.1 × 150 mm, Bonded Silica Products: no. 01425071) column was used for the separation with a flow rate of 0.2 ml/min and an oven temperature of 25°C.

A combination of H_2_O (0.1 vol% formic acid) (A) and acetonitrile (0.1 vol% formic acid) (B) was used as mobile phase and following gradient program was applied: 15 min from 20 to 53 vol% (B), 5 min to 95 vol% (B), 10 min at 95 vol% (B), 1 min to 20 vol% (B), and post-run 10 min 20 vol% (B). All solvents were of HPLC grade, and the method was modified according to Haselmair-Gosch et al. (2018).

Quantification was performed with the following commercially available flavonoid aglyones: apigenin (≥99%, Carl Roth, Germany), butein (≥99%, Extrasynthese, France), dihydrokaempferol (DHK, >98%, TransMIT GmbH, Germany), eriodictyol (≥99%, Extrasynthese, France), formononetin (≥99%, Extrasynthese, France), phloretin (≥99%, Sigma-Aldrich, USA), quercetin (≥99%, Extrasynthese, France), and sulfuretin (≥99%, Extrasynthese, France). Flavonoids were quantified at different wavelengths (290, 309, 340, or 385 nm). Additional investigated aglycones were calculated as equivalents in dependence of their respective type of flavonoid. Further information is provided in Supplementary Table S22. Yields are expressed as mg (μg) flavonoid or flavonoid equivalent per gram wet plant material.

### Identification by UHPLC-ESI-qTOF-MS

Identification of aglycones after enzymatic hydrolysis by snailase was carried out by a 1290 Infinity II LC System equipped with a ZORBAX Eclipse Plus C18 Rapid Resolution HD (1.8 μm, 2.1 × 150 mm) connected to a G7117C diode array detector and subsequently followed by a 6545 LC/Q-TOF (Agilent, USA) with a multimode ion source. The flow rate was set to 0.3 ml/min, and the column oven was operated at 35°C.

The same mobile phase as for HPLC analysis was used with the following gradients, namely, 1 min at 5 vol% (B), 4 min from 5 to 15 vol% (B), 6 min to 53 vol% (B), 4 min to 100 vol% (B), 6.5 min at 100 vol% (B), 0.5 min to 5 vol%, and post run 5 min at 5 vol% (B). The wavelength during analysis was 290 nm. All solvents were of LC-MS grade.

Mass spectrums were recorded in ESI-negative mode between *m/z* 100 and 1,000 Da (scan rate: 2 spectra/s) after a 5 min solvent cut. The mass spectrometer was operated as follows: gas temp.: 350°C, gas flow: 10 l/min, nebulizer: 40 psig, vaporizer: 220°C, VCharge: 1.75 kV, VCap: 1 kV, fragmentor: 180 V, and skimmer: 75 V.

Flavonoid aglycones were identified according to the retention time, substance-specific UV absorbance, and the accurate mass of the molecular ion. All identified flavonoid aglycones and relevant data per plant extract by mass spectrometric analysis are presented in Supplementary Table S23.

### Statistical Analysis

Statistical data analysis was carried out with an Origin 2021 and one-way ANOVA for multiple groups, followed by Tukey's honestly significant difference *post-hoc* test at the 0.05 significance level.

## Results and Discussion

Snailase has been utilized for the hydrolysis of the flavonol glycoside quercetin-3-*O*-rutinoside (Wang et al., 2011), Epimedium flavonoids (Jin et al., 2013; Liu et al., 2019), or isoflavonoids (Wang et al., 2019); however, was not yet applied for flavonoid glycosides from plant extracts. Cellulase, pectinase, as well as glucosidases, were reported for the deglycosylation of flavonoids from plant extracts (Harborne, 1965; Mandalari et al., 2006; Freischmidt et al., 2015). In this study, we compared the efficiency of enzymatic hydrolysis of various flavonoids with the enzyme mix snailase (experiment entries: **Sna1-8**) with that performed with four other enzymes, namely, cellobiase (**Cbi1-5**), cellulase (**Cel1-5**), β-glucosidase (**Glu1-5**), and pectinase (**Pec1-5**). The hydrolysis with each enzyme was optimized by varying pH, temperature, and incubation time. The starting conditions for the enzymatic hydrolysis were selected according to the supplier (see Section Enzymatic and Acidic Hydrolysis). In addition, acidic hydrolysis with HCl (**HCl1-7**) was performed as a reference hydrolysis and optimized at different temperatures, acid molarity, and incubation times. Nine plant species were used as flavonoid sources to include a broad spectrum of flavonoid classes, as well as different matrices. Thus, we used flower petals and leaves of woody as well as non-woody plants. The efficiency during the optimization process was evaluated by the flavonoid yields, expressed as the sums by flavonoid type. For instance, the identified chalcones in *B. ferulifolia*, butein, lanceoletin, and okanin were expressed as *Σ*(chalcones). An overview of all identified flavonoids is given in Figure 1. The optimization of the enzyme mix snailase is described in detail in Section Optimization of flavonoid Glycoside Hydrolysis by Snailase and for the other tested enzymes, as well as acidic hydrolyses, the results are presented in Supplementary Tables S1 to S12.

### Optimization of Flavonoid Glycoside Hydrolysis by Snailase

Herein, the optimization of hydrolysis by snailase of four flower extracts, as well as five leaf extracts, is described. The influence of pH, time, and temperature during hydrolysis, as well as the amount of enzyme, is discussed (Supplementary Tables S1, S2).

As a starting point for the optimization, a temperature of 37°C and pH 6.5 were selected, as recommended by the supplier of snailase. However, there was no specification for the amount of snailase to be used in the assay. Therefore, the deglycosylation was performed with each, a total amount of 2 mg (**Sna1**) and 5 mg (**Sna2**) of snailase powder per assay.

For all analyzed flower extracts, comparable yields of investigated flavonoids can be reported for **Sna1** and **Sna2** at pH 6.5 (Supplementary Table S1). However, the addition of higher amounts of snailase led to significantly higher yields of flavonoid aglycones in some of the leaf extracts, for instance, 19% more *Σ*(flavones) as well as 76% more *Σ*(flavanones) in extracts from *M*. × *piperita*, 46% more *Σ*(flavonols) from *F. sylvatica*, 20% more *Σ*(flavonols) from *M*. × *domestica*, and ultimately, 52% more *Σ*(flavones) from *R. pseudoacacia* were obtained in experiments **Sna2** compared with **Sna1** (Supplementary Table S2, *p* < 0.05). Additionally, in preliminary studies the hydrolysis was carried out with 7 mg of snailase per assay, which did not lead to higher amounts of total flavonoids for the tested plant extracts (data not shown). Hence for subsequent experiments **Sna3-Sna8**, a total amount of 5 mg snailase was selected as higher yields of aglycones were determined.

Subsequently, the pH was lowered to pH 6.0 in **Sna3**. In comparison with **Sna2**, a significant increase of investigated flavonoids can only be reported in leaf extracts of woody plants, namely, 30% more *Σ*(flavonols) yields from *F. sylvatica* and 116% more *Σ*(DHC, dihydrochalcones) from *M*. × *domestica* were observed (Supplementary Table S2, *p* < 0.05). In addition, the influence of temperature at pH 6.0 was investigated by lowering the temperature from 37°C in **Sna3** to 25°C in **Sna4**. The yields of aglycones in flower extracts were in good agreement for both temperatures, but at 25°C significantly less flavonoid aglycones for the following woody-plant extracts were obtained: 23% less *Σ*(flavonols) from *F. sylvatica*, 36% less *Σ*(DHC) from *M*. × *domestica*, and 16% less Σ(flavones) from *R. pseudoacacia* (Supplementary Table S2, *p* < 0.05). Furthermore, the incubation time was reduced from 25 min at pH 6.0 and 37°C in **Sna3** to 10 min in **Sna5**. Most plant extracts showed comparable results for both investigated incubation periods. However, a shortened time in **Sna5** led to a significant reduction of 27% *Σ*(flavanones) in *M*. × *piperita*, 13% *Σ*(flavonols) in *F. sylvatica*, and 17% *Σ*(DHC) in *M*. × *domestica*, respectively (Supplementary Tables S1, S2, *p* < 0.05). Hence, **Sna6-Sna8** were performed at the optimized temperature of 37°C and incubation time of 25 min.

**Figure 2:**
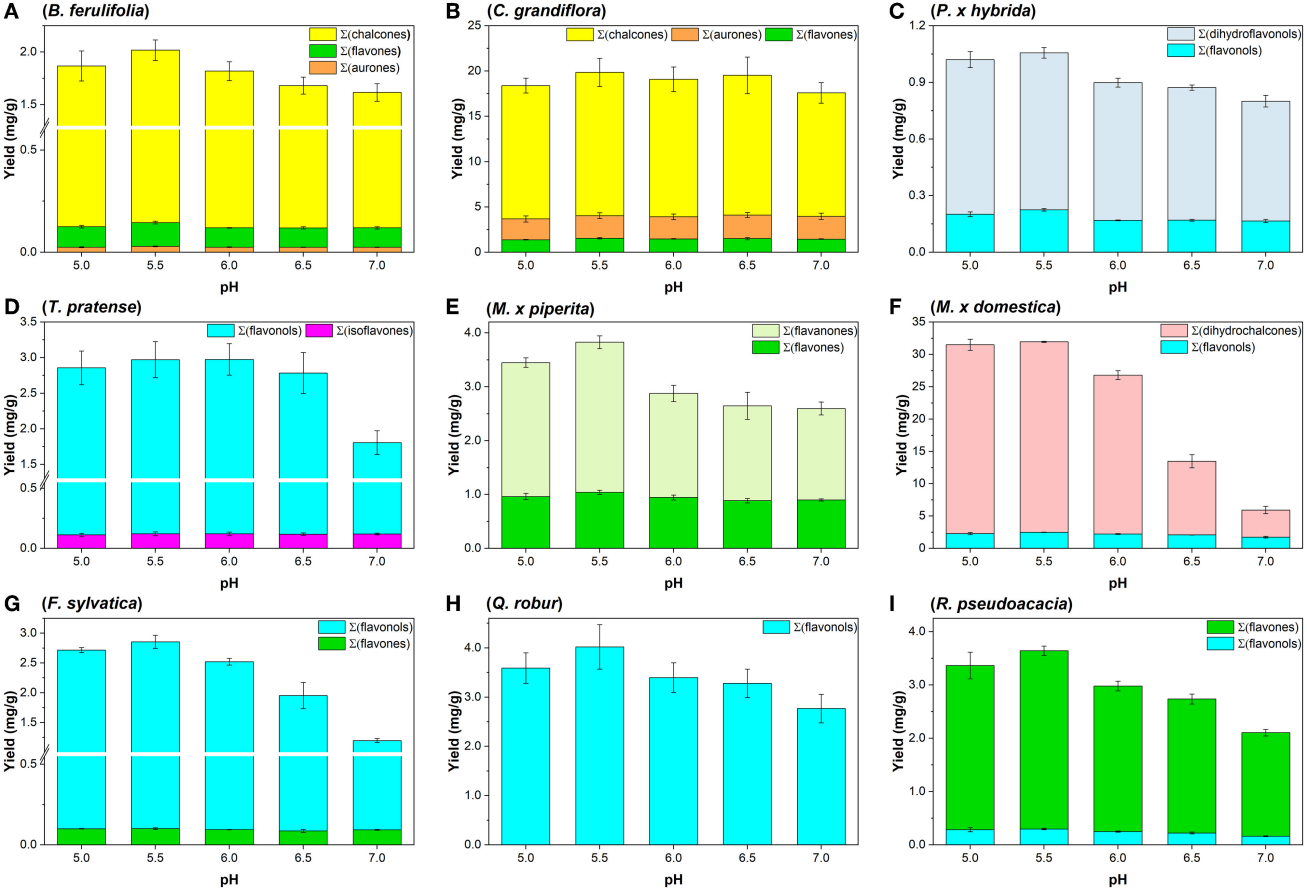
Summarized aglycone yields (mg/g) in methanolic plant extracts after hydrolysis by snailase (5 mg per assay) at 37°C for 25 min between pH 5.0–7.0. **(A)** *B. ferulifolia*; **(B)** *C. grandiflora*; **(C)** *P*. × *hybrida*; **(D)** *T. pratense*; **(E)** *M*. × *piperita*; **(F)** *M*. × *domestica*; **(G)** *F. sylvatica*; **(H)** *Q. robur*; **(I)** *R. pseudoacacia* (*n* = 3 ± SD).

Ultimately, the yields of flavonoid aglycones were investigated additionally at pH 5.0 (**Sna6**), pH 5.5 (**Sna7**), and pH 7.0 (**Sna8)**. For the methanolic flower extracts, flavonoid yields behaved differently in dependence of pH and raw material. First, in *B. ferulifolia* extracts, a maximum yield of *Σ*(flavones) at pH 5.5 in **Sna7**, a significant decrease of *Σ*(chalcones) at pH ≥ 6.5 (*p* < 0.05), and no differences in *Σ*(aurones) yields can be reported (Figure 2A). In comparison, in *C. grandiflora* extracts, where the same flavonoid classes are present as in *B. ferulifolia*, no differences in flavonoid yields were determined within the tested pH range (Figure 2B). Further, *Σ*(DHF, dihydroflavonols) yields from *P*. × *hybrida* were, by a significant margin, the highest at pH ≤ 5.5 as was *Σ*(flavonols) at pH 5.5 (Figure 2C, *p* < 0.05). For *T. pratense*, significantly lower *Σ*(flavonols) yields were only obtained at pH 7 in **Sna8** (Figure 2D, *p* < 0.05) and no changes for *Σ*(isoflavones) yields between pH 5.5–7.0 were observed.

Apart from that, four leaf extracts, of non-woody and woody plants, were investigated. Methanolic leaf extracts of the herb *M*. × *piperita* led to significantly higher *Σ*(flavanones) at pH ≤ 5.5 and *Σ*(flavones) at pH ≤ 6.0 (Figure 2E, *p* < 0.05). Further analysis of tree leaf extracts showed that significantly higher *Σ*(flavonols) values were gained at pH ≤ 6.0 from *M*. × *domestica* (Figure 2F) and *R. pseudoacacia* (Figure 2I). In addition, significantly higher yields of *Σ*(DHC) from *M*. × *domestica*, *Σ*(flavones) from *R. pseudoacacia*, and *Σ*(flavonols) from *F. sylvatica* (Figure 2G) were obtained at pH ≤ 5.5 (*p* < 0.05). However, the amount of *Σ*(flavones) in *F. sylvatica* was comparable for the entire pH range tested. Ultimately, in the extracts of *Q. robur* a significant increase of *Σ*(flavonols) was determined at pH < 7.0 (Figure 2H, *p* < 0.05).

The results indicate that a successful flavonoid glycoside hydrolysis by snailase depends on the experimental parameters, namely, amount of enzyme, temperature, time, and pH. In addition, the type of raw material has an impact on the tested experimental parameters. For instance, yields of *C. grandiflora* were consistent for all tested conditions, but for other flower or leaf extracts, the selection of hydrolysis parameters affected the yields greatly (Supplementary Tables S1, S2). Hence, after the optimization the most effective hydrolysis condition, which led to highest yields of all flavonoids for the investigated plant extracts, consisted of 5 mg enzyme per assay, a temperature of 37°C at pH 5.5 for 25 min in experiment **Sna7**.

### Comparison of Snailase With Other Enzymes and Acidic Hydrolysis

The yields of individual aglycones after enzymatic hydrolysis by snailase are compared with those of the enzymes, cellobiase, cellulase, β-glucosidase, pectinase, and acidic hydrolysis to obtain information about the specificity and efficiency of the deglycosylation of different flavonoid types from several plant extracts.

Flower extracts of *B. ferulifolia* and *C. grandiflora* were used to test the ability of the enzymes to hydrolyze anthochlors. The presence of high levels of different chalcones, as well as contents of aurones and flavones, has been reported in these two plants (Crawford and Smith, 1980; Miosic et al., 2013; Molitor et al., 2015; Boucherle et al., 2017).

**Figure 3:**
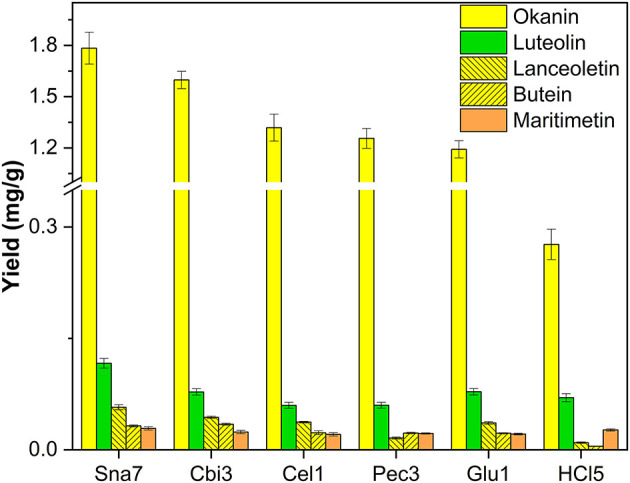
Aglycone yields (mg/g) from flower extracts of *B. ferulifolia*. Enzymatic hydrolysis (25 min): Sna7: 5 mg snailase (pH 5.5, 37°C); Cbi3: 5 U cellobiase (pH 4.5, 37°C); Cel1: 20 U cellulase (pH 5.5, 52°C); Pec3: 20 U pectinase (pH 4.5, 40°C); Glu1: 20 U β-glucosidase (pH 4.5, 37°C). Acidic hydrolysis (60 min): HCl5: 1 M HCl at 100°C (*n* = 3 ± SD).

In the hydrolyzed flower extracts of *B. ferulifolia*, three chalcones, namely, okanin, lanceoletin, and butein, as well as the aurone maritimetin and the flavone luteolin could be identified. Snailase yielded 1.78 mg/g okanin, 0.116 mg/g luteolin, and 57 μg/g lanceoletin, outperforming all other types of hydrolysis significantly (Figure 3; Supplementary Table S13, *p* < 0.05). For butein, both snailase (32.1 μg/g) and cellobiase (34.4 μg/g) gave comparable yields. Furthermore, acidic hydrolysis yielded low yields of the investigated chalcones at the tested conditions; however, comparable yields of maritimetin (26.8 μg/g) to the ones obtained by snailase (28.8 μg/g) were hydrolyzed.

**Figure 4:**
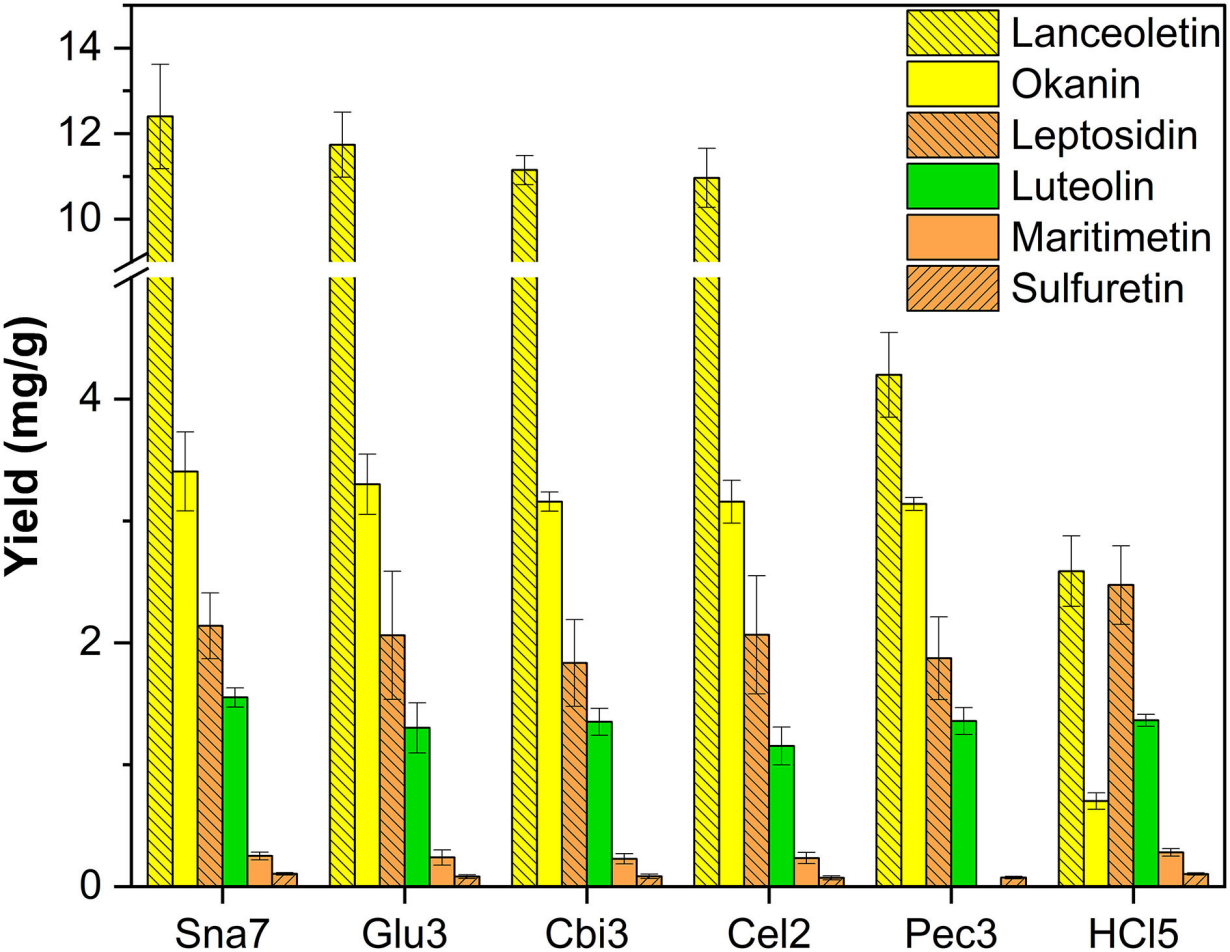
Aglycone yields (mg/g) from flower extracts of *C. grandiflora*. Enzymatic hydrolysis (25 min): Sna7: 5 mg snailase (pH 5.5, 37°C); Glu3: 20 U β-glucosidase (pH 5.5, 37°C); Cbi3: 5 U cellobiase (pH 4.5, 37°C); Cel2: 20 U cellulase (pH 6.0, 52°C); Pec3: 20 U pectinase (pH 4.5, 40°C). Acidic hydrolysis (60 min): HCl5: 1 M HCl at 100°C (*n* = 3 ± SD).

In the extracted flower petals of *C. grandiflora*, derivatives of chalcones (lanceoletin, okanin), aurones (leptosidin, maritimetin, sulfuretin), and luteolin were present. In contrast to the results of *B. ferulifolia* extracts, all enzymes yielded comparable amounts of okanin (3.1–3.4 mg/g) (Figure 4; Supplementary Table S14). The yields of the predominantly accumulated chalcone, lanceoletin, were in good agreement for snailase, cellobiase, cellulase, and β-glucosidase, ranging from 11.0 to 12.4 mg/g, but not for pectinase and acidic hydrolysis, which yielded significantly less, ranging from 2.6 to 4.2 mg/g (*p* < 0.05). Hence, pectinase seems to hydrolyze okanin better than lanceoletin glycosides, from *C. grandiflora* extracts. The enzyme cellulase yielded less luteolin (1.15 mg/g), but results of the other hydrolysis methods were in good agreement (1.30–1.36 mg/g) compared with snailase (1.55 mg/g). Additionally, three aurones, maritimetin (0.23–0.28 mg/g), leptosidin (1.8–2.5 mg/g), and sulfuretin (71–104 μg/g), were quantified in comparable amounts for all tested hydrolysis, apart from pectinase, where no maritimetin could be detected, due to peak interferences. It should be noted that acidic hydrolysis led to high amounts of aurone aglycones, but to low yields of chalcone aglycones, due to the possible isomerization to flavanones in acidic milieu (Cisak and Mielczarek, 1992). Hence, acidic hydrolysis is not recommended to quantify chalcone aglycones from plant extracts.

Flower extracts extracts of *P*. × *hybrida* were included in the study as a source of flavonols and DHFs. The latter are intermediates in the formation of anthocyanins and, therefore, rarely accumulate in plants. However, the escaped genetically modified orange petunias were recently described to contain high amounts of DHK because they possess a rare biochemical background in which flavonol synthase activity is low and dihydroflavonol 4-reductase possesses a very low specificity for DHK compared with other DHFs (Haselmair-Gosch et al., 2018).

**Figure 5:**
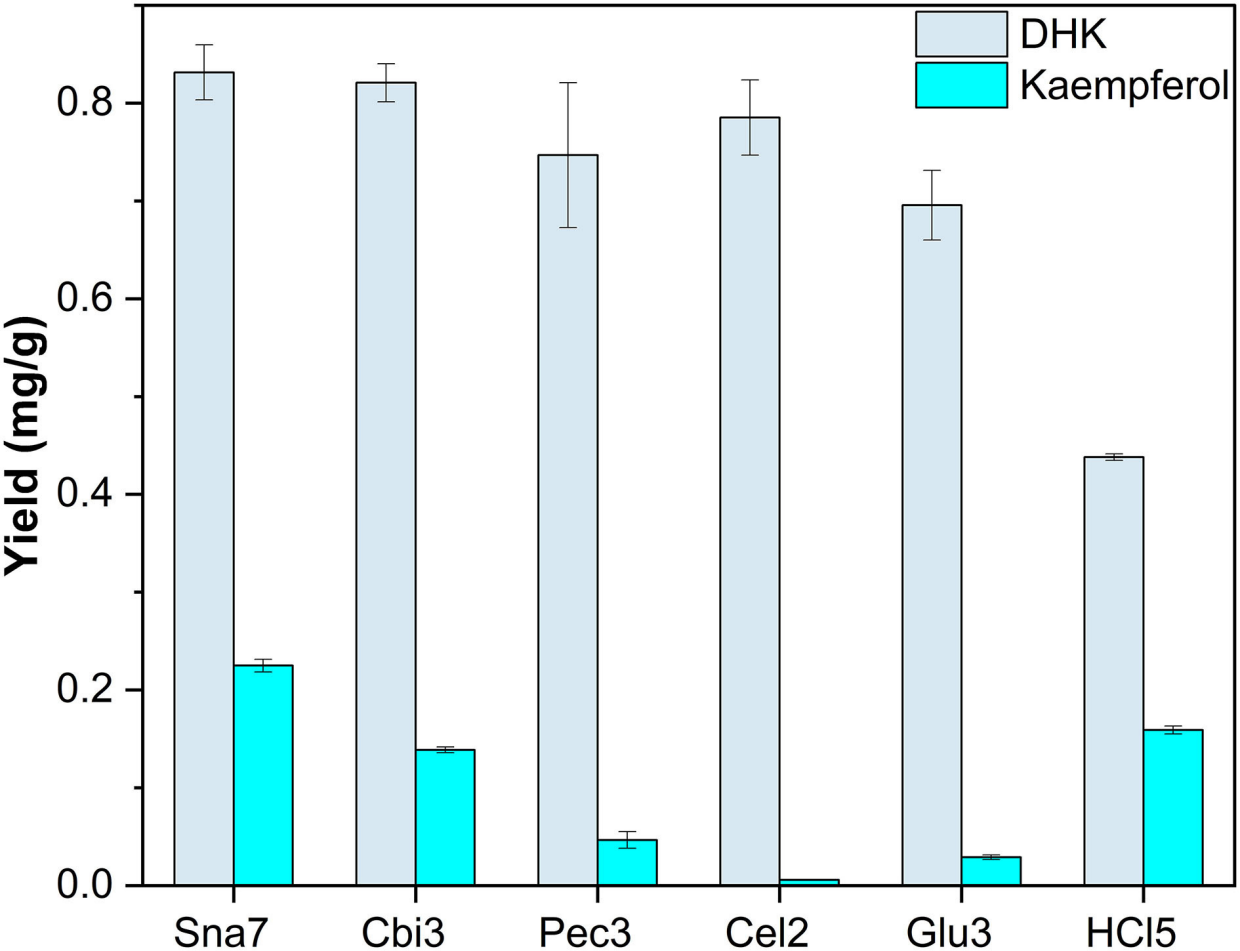
Aglycone yields (mg/g) from flower extracts of *P*. × *hybrida*. Enzymatic hydrolysis (25 min): Sna7: 5 mg snailase (pH 5.5, 37°C); Cbi3: 5 U cellobiase (pH 4.5, 37°C); Pec3: 20 U pectinase (pH 4.5, 40°C); Glu3: 20 U β-glucosidase (pH 5.5, 37°C); Cel2: 20 U cellulase (pH 6.0, 52°C). Acidic hydrolysis (60 min): HCl5: 1 M HCl at 100°C (*n* = 3 ± SD).

Snailase, cellulase, cellobiase, and pectinase yielded significantly higher amounts of DHK, ranging from 0.75 to 0.831 mg/g compared with the rest (Figure 5; Supplementary Table S15, *p* < 0.05). Furthermore, hydrolysis with snailase yielded the highest amounts of kaempferol (0.225 mg/g). Although the other three aforementioned enzymes yielded comparable amounts of DHK, significantly less kaempferol by 38 up to 97% was obtained compared with snailase (*p* < 0.05).

**Figure 6:**
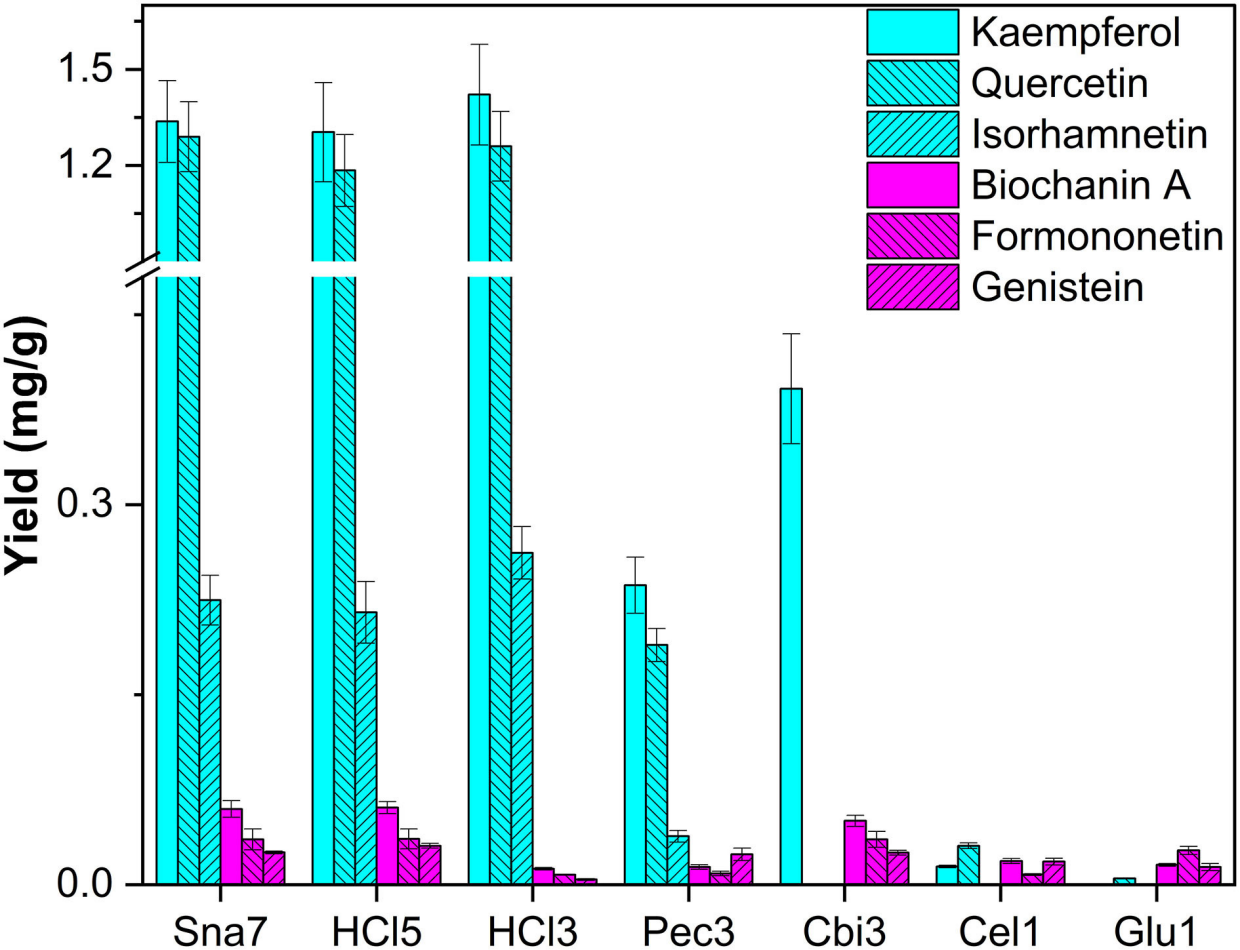
Aglycone yields (mg/g) from flower extracts of *T. pratense*. Enzymatic hydrolysis (25 min): Sna7: 5 mg snailase (pH 5.5, 37°C); Pec3: 20 U pectinase (pH 4.5, 40°C), Cbi3: 5 U cellobiase (pH 4.5, 37°C); Cel1: 20 U cellulase (pH 5.5, 52°C); Glu1: 20 U β-glucosidase (pH 4.5, 37°C). Acidic hydrolysis (60 min): HCl3: 1 M HCl at 70°C; HCl5: 1 M HCl at 100°C (*n* = 3 ± SD).

The last flower model, *T. pratense*, was selected for extraction, primarily due to the content of glycosylated isoflavones (biochanin A, formononetin, genistein), among other flavonols, e.g., kaempferol, isorhamnetin, and quercetin (Lin et al., 2000). Deglycosylation by snailase and HCl (70° and 100°C, respectively) led to higher yields of investigated flavonols (1.30–1.42 mg/g kaempferol, 1.18–1.29 mg/g quercetin, 0.215–0.262 mg/g isorhamnetin) compared with all other tested enzymes (Figure 6; Supplementary Table S16, *p* < 0.05). In addition, a particular group of flavonoids, isoflavones, could be investigated in *T. pratense*. Biochanin A (60–61 μg/g), formononetin (36 μg/g), and genistein (25.4–30.7 μg/g) were generated to the greatest extent by snailase and HCl (100°C). Furthermore, enzymatic hydrolysis by cellobiase led to comparable yields of all investigated isoflavones compared with those by snailase, but it was not able to hydrolyze flavonol glycosides sufficiently (Figure 6). Apart from that, the yields of isoflavones were significantly different between acidic hydrolysis at 70 and 100°C. In total, 79% less biochanin A, 78% less formononetin, and 87% less genistein were obtained at lower temperatures (*p* < 0.05). However, both tested acidic hydrolyses yielded high amounts of flavonols, implying that the optimal conditions for the simultaneous acidic hydrolysis of different flavonoid types needs to be optimized carefully. Different acidic hydrolysis optima in dependence of the flavonoid class in one plant have been discussed before (Nuutila et al., 2002).

**Figure 7:**
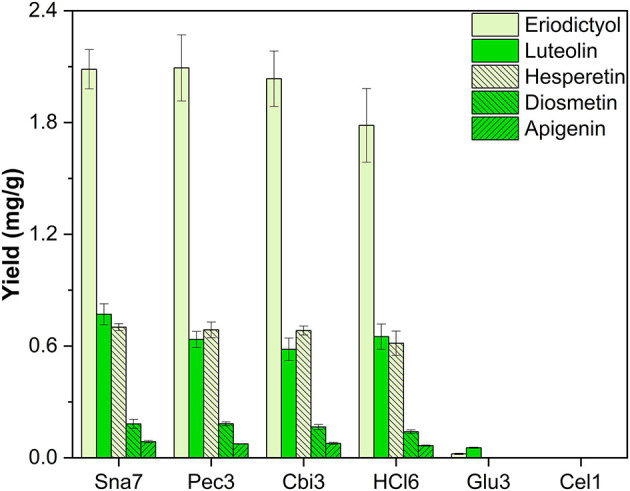
Aglycone yields (mg/g) from leaf extracts of *M*. × *piperita*. Enzymatic hydrolysis (25 min): Sna7: 5 mg snailase (pH 5.5, 37°C); Pec3: 20 U pectinase (pH 4.5, 40°C); Cbi3: 5 U cellobiase (pH 4.5, 37°C); Glu3: 20 U β-glucosidase (pH 5.5, 37°C); Cel1: 20 U cellulase (pH 5.5, 52°C). Acidic hydrolysis (60 min): HCl5: 1 M HCl at 100°C (*n* = 3 ± SD).

Besides the investigated flower extracts, the leaves of five plants were extracted with methanol. Leaf extracts of the herb *M*. × *piperita* were analyzed due to their accumulation of flavanone (eriodictyol, hesperetin) and flavone (apigenin, diosmetin, luteolin) glycosides (Inoue et al., 2002; Kapp et al., 2013). Hydrolysis by snailase, cellobiase, pectinase, and HCl resulted in the highest yields, by a significant margin, of the flavananones, eriodictyol ranging from 1.78 to 2.09 mg/g and hesperetin from 0.61 to 0.701 mg/g (Figure 7; Supplementary Table S17, *p* < 0.05).

Further investigation of the flavone yields showed that snailase produced high yields of luteolin (0.77 mg/g), diosmetin (0.182 mg/g), and apigenin (86 μg/g). Flavone yields by cellobiase, pectinase, and HCl correlated to some of the flavone yields by snailase, but, importantly, were not able to generate all three flavones at the same time in highest quantities. Enzymatic hydrolyses by β-glucosidase and cellulase were not feasible for the target aglycones accumulated in *M*. × *piperita* (Figure 7).

**Figure 8:**
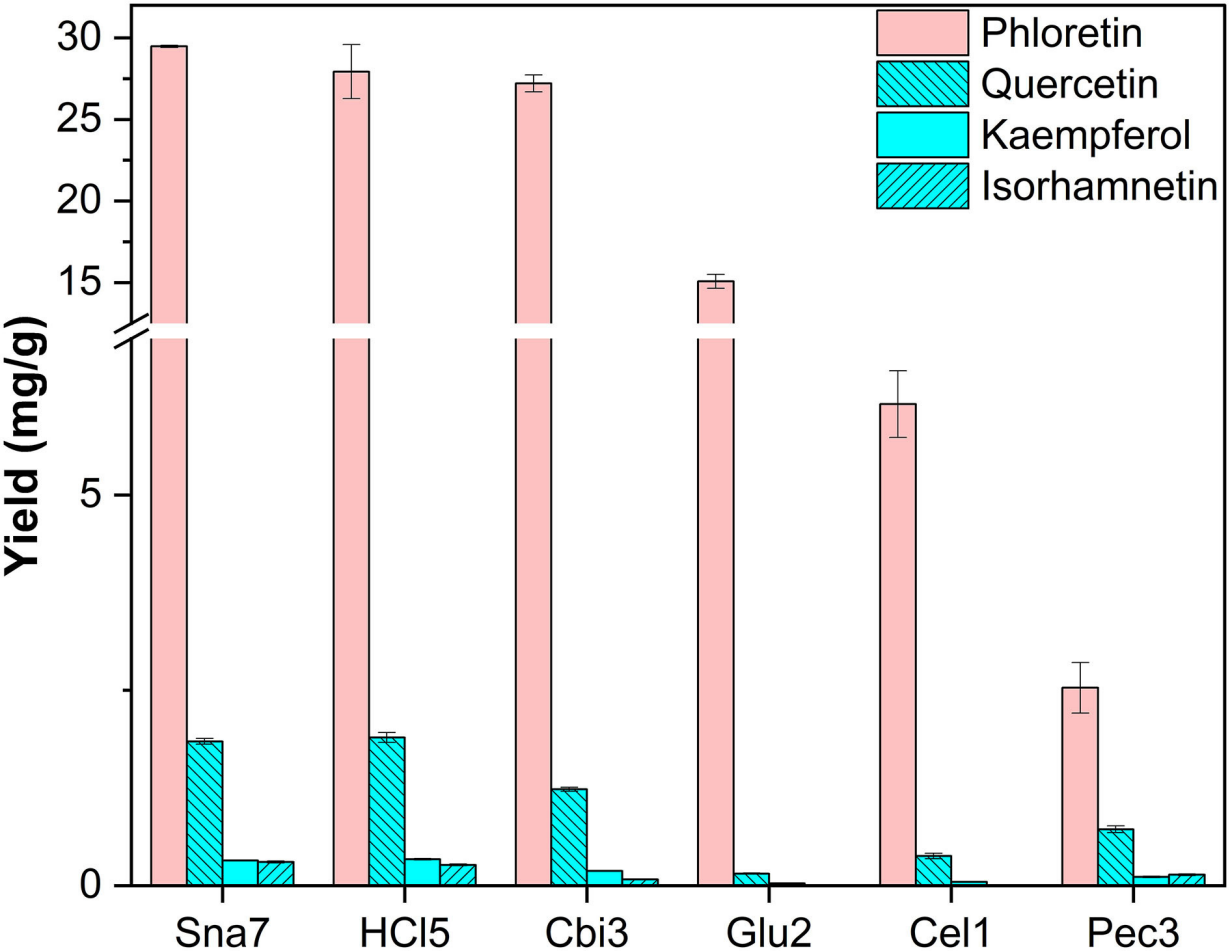
Aglycone yields (mg/g) from leaf extracts of *M*. × *domestica*. Enzymatic hydrolysis (25 min): Sna7: 5 mg snailase (pH 5.5, 37°C); Cbi3: 5 U cellobiase (pH 4.5, 37°C); Glu2: 20 U β-glucosidase (pH 5.0, 37°C); Cel1: 20 U cellulase (pH 5.5, 52°C); Pec3: 20 U pectinase (pH 4.5, 40°C). Acidic hydrolysis (60 min): HCl5: 1 M HCl at 100°C (*n* = 3 ± SD).

Additionally, leaves of four trees were investigated. First, the leaves of *M*. × *domestica* were included as an exceptionally good source for DHC glycosides. In accordance with the literature, derivatives of the dihydrochalcone phloretin, as well as the flavonols quercetin, kaempferol, and isorhamnetin, were found (Bernonville et al., 2011). After acidic hydrolysis (100°C), the most abundant aglycone present was phloretin, which was yielded in the highest amounts, significantly so, by snailase (29.48 mg/g) and HCl (27.9 mg/g), compared with the other tested enzymes (Figure 8; Supplementary Table S18, *p* < 0.05). In addition, hydrolysis by HCl and snailase led to the highest amounts of the flavonols, quercetin, ranging from 1.85 to 1.90 mg/g, and kaempferol, 0.321 to 0.337 mg/g (*p* < 0.05). Ultimately, snailase generated the highest quantities of isorhamnetin (0.302 mg/g, *p* < 0.05).

**Figure 9:**
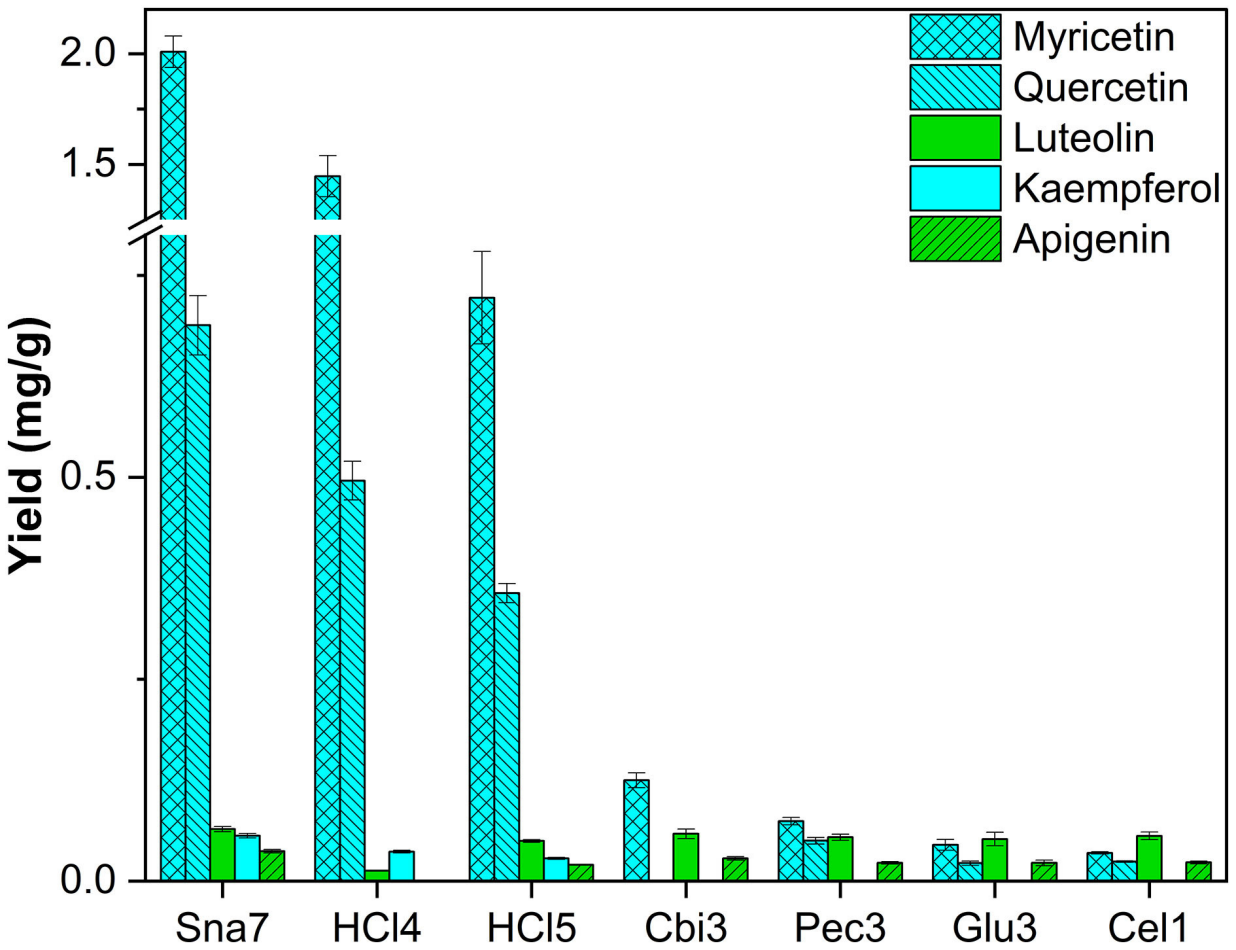
Aglycone yields (mg/g) from leaf extracts of *F. sylvatica*. Enzymatic hydrolysis (25 min): Sna7: 5 mg snailase (pH 5.5, 37°C); Cbi3: 5 U cellobiase (pH 4.5, 37°C); Pec3: 20 U pectinase (pH 4.5, 40°C); Glu3: 20 U β-glucosidase (pH 5.5, 37°C); Cel1: 20 U cellulase (pH 5.5, 52°C). Acidic hydrolysis (60 min): HCl4: 2 M HCl at 70°C HCl5; 1 M HCl at 100°C (*n* = 3 ± SD).

Leaf extracts of *F. sylvatica* were analyzed and three flavonols (myricetin, kaempferol, and quercetin) and two flavones (apigenin and luteolin) were characterized after removal of the sugar moieties. These flavonoids have been known to accumulate in *F. sylvatica* (Pirvu et al., 2013; Formato et al., 2021). For all flavonols, snailase outperformed the other enzymes significantly, yielding 2.01 mg/g myricetin, 0.69 mg/g quercetin, and 56.1 μg/g kaempferol (Figure 9; Supplementary Table S19, *p* < 0.05). Yields of myricetin were approximately 16 times greater, and those of quercetin 14 times greater, compared to the other enzymes. It has to be noted that of all enzymes only snailase hydrolyzed kaempferol glycosides to the respective aglycone.

Furthermore, all enzymes yielded comparable amounts of luteolin (52–64 μg/g), but for the second flavone present, apigenin, significantly greater quantities (36.9 μg/g) were determined after hydrolysis by snailase (*p* < 0.05). Apart from that, acidic hydrolysis at 70°C (2 M HCl) led to the second highest yields of the three flavonols, but low yields for the target flavones. However, acidic hydrolysis at 100°C (1M HCl) led to higher yields of flavones, but lower yields of flavonols (Supplementary Table S19, *p* < 0.05) in comparison. Hence, an optimal condition for acidic hydrolysis of flavonoids was not observed and different preferable hydrolysis parameters for flavonols and flavones have been discussed before (Ahn-Jarvis et al., 2019).

**Figure 10:**
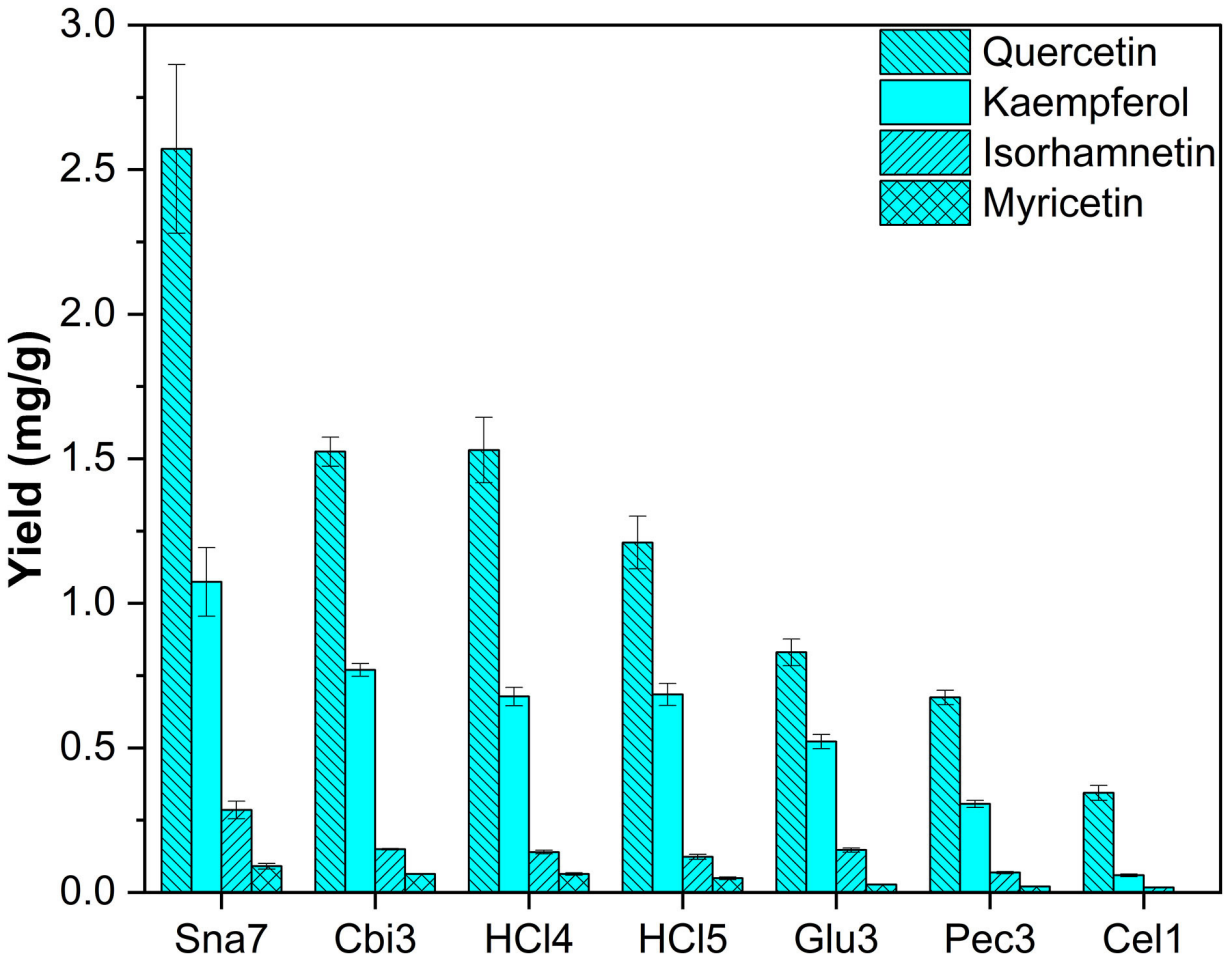
Aglycone yields (mg/g) from leaf extracts of *Q. robur*. Enzymatic hydrolysis (25 min): Sna7: 5 mg snailase (pH 5.5, 37°C); Cbi3: 5 U cellobiase (pH 4.5, 37°C); Glu3: 20 U β-glucosidase (pH 5.5, 37°C); Pec3: 20 U pectinase (pH 4.5, 40°C); Cel1: 20 U cellulase (pH 5.5, 52°C). Acidic hydrolysis (60 min): HCl4: 2 M HCl at 70°C; HCl5:1 M HCl at 100°C (*n* = 3 ± SD).

Leaves of *Q. robur* were included as an additional tree species, which primarily contain flavonols. After hydrolysis, quercetin, kaempferol, myricetin, and isorhamnetin were found as reported in literature (Tikkanen and Julkunen-Tiitto, 2003; Unuofin and Lebelo, 2021). Snailase yielded significantly higher amounts of all flavonol aglycones, for instance, ≥68% more quercetin (2.57 mg/g), ≥39% more kaempferol (1.07 mg/g), ≥94% more isorhamnetin (0.29 mg/g), and ≥41% more myricetin (90 μg/g) were determined compared with other hydrolysis experiments (Figure 10; Supplementary Table S20, *p* < 0.05).

Differences between hydrolysis by acid at 70°C (2 M HCl) and 100°C (1M HCl) can be reported for myricetin. Yields increased significantly with lower temperature and molarity from 49 to 63 μg/g (Supplementary Table S20, *p* < 0.05); however, the other two flavonols present, namely, kaempferol and quercetin, stayed unchanged. In comparison with *F. sylvatica* extracts, where the same flavonols were found as in *Q. robur*, an increase of myricetin yields was also observed, after acidic hydrolysis by 2 M HCl at 70°C, compared with 1 M HCl at 100°C. However, different acidic hydrolysis conditions led to changes in kaempferol and quercetin yields (Supplementary Table S19). Hence, the yields of flavonoid aglycones by acidic hydrolysis seem to depend not only on the hydrolysis conditions but also on the nature of the plant, which has been the object of discussion (Nuutila et al., 2002).

**Figure 11:**
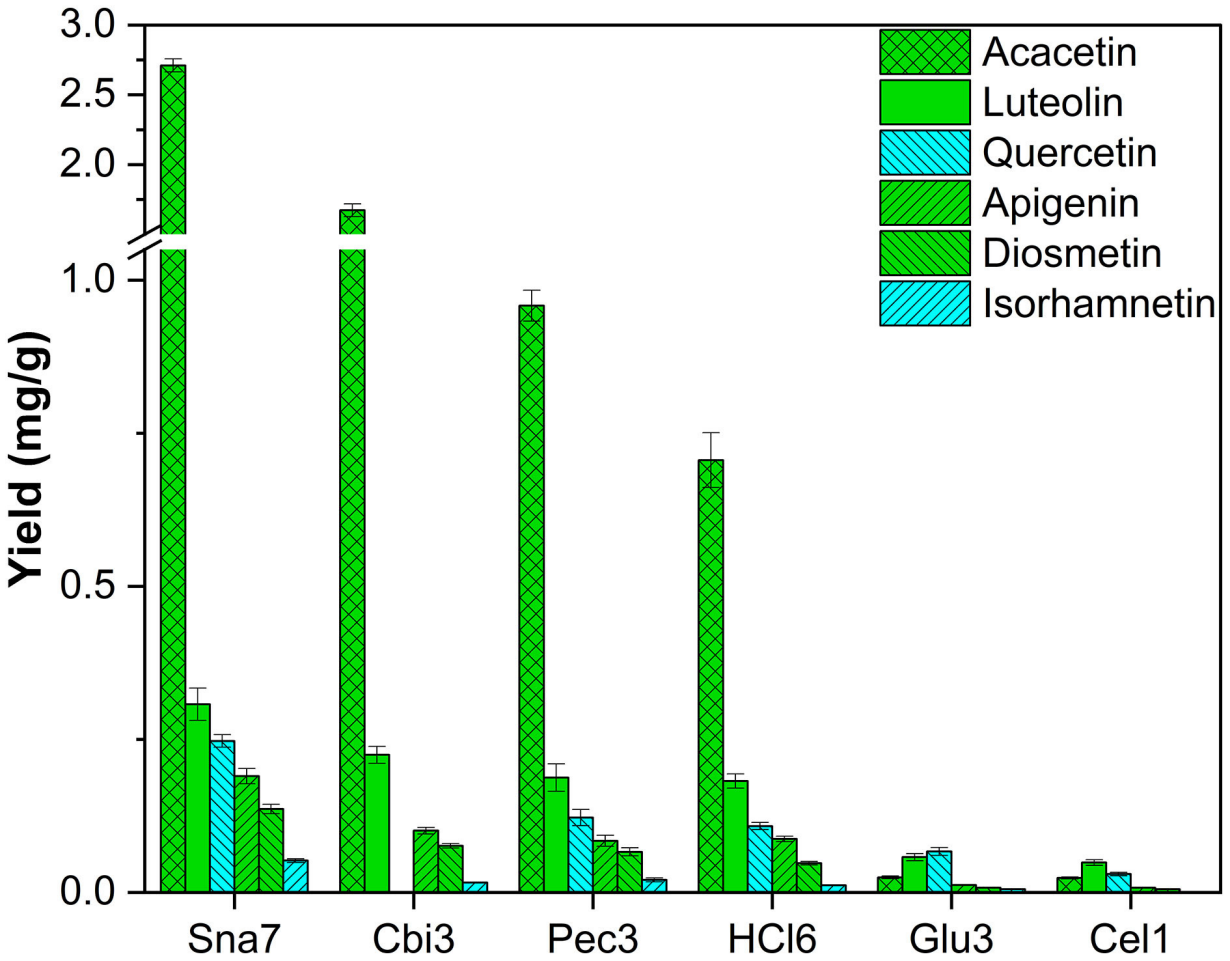
Aglycone yields (mg/g) from leaf extracts of *R. pseudoacacia*. Enzymatic hydrolysis (25 min): Sna7: 5 mg Snailase (pH 5.5, 37°C); Cbi3: 5 U Cellobiase (pH 4.5, 37°C); Pec3: 20 U Pectinase (pH 4.5, 40°C); Glu3: 20 U β-Glucosidase (pH 5.5, 37°C); Cel1: 20 U Cellulase (pH 5.5, 52°C). Acidic hydrolysis (60 min): HCl6: 2M HCl at 100°C (*n* = 3 ± SD).

Ultimately, methanolic extracts of *R. pseudoacacia* leaves were investigated. In total, derivatives of six flavonoids were determined, namely, four flavones (acacetin, apigenin, diosmetin, and luteolin) and two flavonols (isorhamnetin and quercetin). Several glycosides of these flavonoids have been reported (Veitch et al., 2010). Aglycone yields by snailase were significantly higher than those of all other investigated hydrolysis methods (Figure 11; Supplementary Table S21, *p* < 0.05). In total, ≥62% more acacetin (2.71 mg/g), ≥36% more luteolin (0.307 mg/g), ≥102% more quercetin (0.247 mg/g), ≥88% more apigenin (0.190 mg/g), ≥79% more diosmetin (0.136 mg/g), and ≥160% more isorhamnetin (52 μg/g) were hydrolyzed in comparison to all other types of hydrolysis.

Enzymatic hydrolysis by cellobiase, cellulase, β-glucosidase, and pectinase showed different behavior, and the results indicate that the success of flavonoid deglycosylation depends on several parameters, e.g., selection of enzyme, type of plant, as well as type of flavonoid. However, the enzyme mix snailase yielded highest amounts of all target flavonoids independently of type of plant or flavonoid.

By comparing the amounts of the two most abandoned classes of flavonoids, flavonols, and flavones, each found in five plant extracts, several observations can be made. Snailase outperformed all other enzymes by yielding significantly higher amounts of all targeted flavonols (isorhamnetin, myricetin, kaempferol, and quercetin). In contrast, flavone yields differ in the results, depending on the type of plant and flavonoid. For instance, luteolin was yielded in the highest amounts by several enzymes in *C. grandiflora* and *F. sylvatica*, as with snailase, but significantly higher yields were hydrolyzed by snailase in extracts of *B. ferulifolia* and *M*. × *piperita* in comparison to other enzymes (*p* < 0.05). Similar behavior can be observed for anthochlors' aglycones. Okanin, lanceoletin, and maritimetin yields generated by several enzymes in *C. grandiflora* (Supplementary Table S14) correlated with the amounts obtained by snailase. However, in *B. ferulifolia*, where the same anthochlors were determined, the other enzymes led to significantly lower yields than snailase (Supplementary Table S13). This could indicate that the success of hydrolysis depends on the combination of enzyme and type of plant. Differences in aglycone yields in dependence of the enzyme have been reported before by comparing the amounts of hydrolyzed flavonoids in bergamot peel extracts yielded by cellulase or pectinase (Mandalari et al., 2006).

By focusing on one plant, differences in yields in dependence of flavonoid classes can be reported. For instance, in *P*. × *hybrida* extracts, the dihydroflavonol DHK was hydrolyzed in high yields by cellobiase or cellulase, but both enzymes generated significantly lower yields of the flavonol kaempferol than snailase, although the structures of both polyphenols are similar (Figure 5). Furthermore, differences in enzyme selectivity within one flavonoid classes can also be reported. Cellobiase and β-glucosidase in *T. pratense* led to high yields of formononetin, but to low amounts of biochanin A (Figure 6). Apart from that, high yields of luteolin were generated by all tested enzymes in *F. sylvatica* extracts; however, the other present flavone, apigenin, was yielded in highest amounts by snailase alone (Figure 9). Interestingly, pectinase hydrolyzed highest amounts of apigenin in *M*. × *piperita* extracts (Supplementary Table S17). This could indicate the molecular structure of the flavonoid plays a minor role and possibly the type and binding of the sugar moiety could affect the success of hydrolysis. The binding site of *O*-glycosidic bonds has been reported to potently influence the hydrolyzability of flavonoid glycosides (Harborne, 1965).

Ultimately, results of acidic hydrolysis by HCl depended on the temperature (Supplementary Tables S11, S12), type of plant, and flavonoid. High yields for some flavonoids were obtained, for instance, flavanones in *M*. × *piperita* extracts, aurones in *C. grandiflora* and *B. ferulifolia*, or phloretin from *M*. × *domestica*, among others. Influences for efficiently hydrolyzing by acid have been discussed before (Nuutila et al., 2002; Ahn-Jarvis et al., 2019) and correlate with the results herein.

## Conclusion

Our study shows that, for all tested methanolic flower extracts (*B. ferulifolia, C. grandiflora, P*. × *hybrida, T. pratense*) and leaf extracts (*F. sylvatica, M*. × *domestica, M*. × *piperita, Q. robur, R. pseudoacacia*) flavonoid hydrolysis was most feasible by the enzyme mix snailase compared with cellobiase, cellulase, β-glucosidase, and pectinase. It is assumed that the success of the enzymatic hydrolysis for flavonoid glycosides depends mainly on four parameters, namely, enzyme selection, optimization of hydrolysis (pH, temperature, and time), type of plant, and type of flavonoid. However, the results indicate that the enzyme mix snailase functions independently of the type of plant and flavonoid as it yielded the highest amounts of all 21 investigated flavonoid aglycones compared with the other tested enzymes. The optimal conditions for flavonoid glycoside hydrolysis by snailase are 37°C, pH 5.5, and 5 mg snailase per assay for 25 min, which are recommended for the screening and determination of contents of flavonoid aglycones from unknown plant samples. Hence, snailase is a useful and promising tool for flavonoid hydrolysis of various plants with numerous flavonoid classes as well as glycosides for comparative studies and routine analysis.

## Data Availability Statement

The original contributions presented in the study are included in the article/Supplementary Material, further inquiries can be directed to the corresponding author/s.

## Author Contributions

CK wrote the original draft. CK and HH conceived the research and wrote the manuscript. CK and JS conducted the experiments and analyzed the data. All authors approved the manuscript.

## Funding

We acknowledge funding by the Austrian Science Fund (FWF) I 2919-B25 and by the Vienna Science and Technology Fund (WWTF, Project Number: ESR17-027). CK acknowledges TU Wien for the funding of the Doctoral College Bioactive (https://bioactive.tuwien.ac.at/home/).

## Conflict of Interest

The authors declare that the research was conducted in the absence of any commercial or financial relationships that could be construed as a potential conflict of interest.

## Publisher's Note

All claims expressed in this article are solely those of the authors and do not necessarily represent those of their affiliated organizations, or those of the publisher, the editors and the reviewers. Any product that may be evaluated in this article, or claim that may be made by its manufacturer, is not guaranteed or endorsed by the publisher.
