# Supplementary material for: Snailase: A Promising Tool for the Enzymatic Hydrolysis of Flavonoid Glycosides From Plant Extracts
Source: Front Plant Sci. 2022 Jun 9;13:889184. doi: 10.3389/fpls.2022.889184 (PMC9218754; doi:10.3389/fpls.2022.889184)
Supplement: Supplementary file 1 [file Data_Sheet_1.docx]

Supplementary Material

Table S1 Summarized aglycone yields by hydrolysis with snailase at various parameters for the methanolic flower extracts (n = 3 ± SD).

| Parameters | | | | | ***B. ferulifolia*** | | | ***C. grandiflora*** | | | ***P. × hybrida*** | | ***T. pratense*** | | |
| --- | --- | --- | --- | --- | --- | --- | --- | --- | --- | --- | --- | --- | --- | --- | --- |
| No. | pH | T  (°C) | t  (min) | m  (mg) | Σ(aurones)  (µg/g) | Σ(chalcones)  (mg/g) | Σ(flavones)  (mg/g) | Σ(aurones)  (mg/g) | Σ(chalcones)  (mg/g) | Σ(flavones)  (mg/g) | Σ(DHF)  (mg/g) | Σ(flavonols)  (mg/g) | Σ(flavonols)  (mg/g) | Σ(isoflavones)  (mg/g) | |
| Sna1 | 6.5 | 37 | 25 | 2 | 26.1 ± 1.7^a^ | 1.64 ± 0.07^ab^ | 0.100 ± 0.005^b^ | 2.4 ± 0.3^a^ | 14.4 ± 0.8^a^ | 1.41 ± 0.03^a^ | 0.718 ± 0.007^bc^ | 0.1652 ± 0.0029^d^ | 2.24 ± 0.24^ab^ | 0.119 ± 0.012^a^ | |
| Sna2 | 6.5 | 37 | 25 | 5 | 24.7 ± 1.3^a^ | 1.56 ± 0.08^b^ | 0.095 ± 0.006^b^ | 2.59 ± 0.28^a^ | 15.4 ± 2.0^a^ | 1.51 ± 0.12^a^ | 0.703 ± 0.014^cd^ | 0.170 ± 0.005^cd^ | 2.67 ± 0.29^a^ | 0.118 ± 0.011^a^ | |
| Sna3 | 6.0 | 37 | 25 | 5 | 25.1 ± 1.6^a^ | 1.70 ± 0.09^ab^ | 0.0948 ± 0.0018^b^ | 2.4 ± 0.3^a^ | 15.2 ± 1.4^a^ | 1.48 ± 0.04^a^ | 0.729 ± 0.024^bc^ | 0.1689 ± 0.0023^cd^ | 2.85 ± 0.22^a^ | 0.121 ± 0.015^a^ | |
| Sna4 | 6.0 | 25 | 25 | 5 | 25.5 ± 1.2^a^ | 1.70 ± 0.08^ab^ | 0.093 ± 0.004^b^ | 2.46 ± 0.28^a^ | 15.4 ± 1.7^a^ | 1.50 ± 0.09^a^ | 0.728 ± 0.006^bc^ | 0.169 ± 0.004^cd^ | 2.6 ± 0.3^a^ | 0.118 ± 0.011^a^ | |
| Sna5 | 6.0 | 37 | 10 | 5 | 25.8 ± 2.8^a^ | 1.75 ± 0.10^ab^ | 0.097 ± 0.006^b^ | 2.3 ± 0.4^a^ | 14.6 ± 0.9^a^ | 1.38 ± 0.06^a^ | 0.777 ± 0.027^ab^ | 0.189 ± 0.008^bc^ | 2.7 ± 0.3^a^ | 0.122 ± 0.013^a^ | |
| Sna6 | 5.0 | 37 | 25 | 5 | 24.9 ± 1.8^a^ | 1.74 ± 0.14^ab^ | 0.100 ± 0.007^b^ | 2.3 ± 0.3^a^ | 14.7 ± 0.8^a^ | 1.38 ± 0.04^a^ | 0.82 ± 0.04^a^ | 0.201 ± 0.012^b^ | 2.75 ± 0.24^a^ | 0.112 ± 0.014^a^ | |
| Sna7 | 5.5 | 37 | 25 | 5 | 28.8 ± 2.2^a^ | 1.87 ± 0.10^a^ | 0.117 ± 0.006^a^ | 2.5 ± 0.3^a^ | 15.8 ± 1.5^a^ | 1.55 ± 0.08^a^ | 0.831 ± 0.028^a^ | 0.225 ± 0.007^a^ | 2.85 ± 0.25^a^ | 0.121 ± 0.016^a^ | |
| Sna8 | 7.0 | 37 | 25 | 5 | 25.0 ± 1.2^a^ | 1.50 ± 0.08^b^ | 0.095 ± 0.006^b^ | 2.5 ± 0.4^a^ | 13.6 ± 1.1^a^ | 1.46 ± 0.04^a^ | 0.64 ± 0.03^d^ | 0.166 ± 0.009^d^ | 1.69 ± 0.17^b^ | 0.119 ± 0.007^a^ | |
| Mean values with different letters (a, b, etc.) within the same column are statistically different (*p < 0.05*)  DHF: dihydroflavonols | | | | | | | | | | | | | | |  |

Table S2 Summarized aglycone yields by hydrolysis with snailase at various parameters for the methanolic flower extracts (n = 3 ± SD).

| Parameters | | | | | ***F. sylvatica*** | | ***M. × domestica*** | | ***M. × piperita*** | | ***Q. robur*** | ***R. pseudoacacia*** | | |
| --- | --- | --- | --- | --- | --- | --- | --- | --- | --- | --- | --- | --- | --- | --- |
| No. | pH | T  (°C) | t  (min) | m  (mg) | Σ(flavones)  (µg/g) | Σ(flavonols)  (mg/g) | Σ(DHC)  (mg/g) | Σ(flavonols)  (mg/g) | Σ(flavones)  (mg/g) | Σ(flavanones)  (mg/g) | Σ(flavonols)  (mg/g) | Σ(flavones)  (mg/g) | Σ(flavonols)  (mg/g) | |
| Sna1 | 6.5 | 37 | 25 | 2 | 96.1 ± 0.4^ab^ | 1.282 ± 0.004^d^ | 9.6 ± 0.5^e^ | 1.73 ± 0.06^c^ | 0.74 ± 0.06^c^ | 1.00 ± 0.09^d^ | 3.07 ± 0.27^b^ | 1.65 ± 0.06^d^ | 0.173 ± 0.016^cd^ | |
| Sna2 | 6.5 | 37 | 25 | 5 | 87 ± 9^b^ | 1.87 ± 0.22^c^ | 11.4 ± 1.0^e^ | 2.082 ± 0.019^b^ | 0.88 ± 0.04^b^ | 1.76 ± 0.25^bc^ | 3.28 ± 0.29^ab^ | 2.51 ± 0.09^bc^ | 0.223 ± 0.018^bc^ | |
| Sna3 | 6.0 | 37 | 25 | 5 | 94.2 ± 1.1^ab^ | 2.43 ± 0.06^b^ | 24.6 ± 0.7^b^ | 2.22 ± 0.07^ab^ | 0.94 ± 0.05^ab^ | 1.93 ± 0.15^b^ | 3.4 ± 0.3^ab^ | 2.73± 0.09^b^ | 0.249 ± 0.011^ab^ | |
| Sna4 | 6.0 | 25 | 25 | 5 | 99 ± 3^ab^ | 1.86 ± 0.04^c^ | 15.7 ± 1.7^d^ | 2.28 ± 0.04^ab^ | 0.941 ± 0.007^ab^ | 1.83 ± 0.09^b^ | 3.44 ± 0.08^ab^ | 2.29 ± 0.07^c^ | 0.218 ± 0.020^bcd^ | |
| Sna5 | 6.0 | 37 | 10 | 5 | 96 ± 5^ab^ | 2.12 ± 0.15^c^ | 20.4 ± 2.3^c^ | 2.24 ± 0.11^ab^ | 0.91 ± 0.04^b^ | 1.41 ± 0.04^c^ | 3.4 ± 0.3^ab^ | 2.681 ± 0.026^b^ | 0.227 ± 0.014^bc^ | |
| Sna6 | 5.0 | 37 | 25 | 5 | 99.9 ± 1.9^a^ | 2.62 ± 0.04^ab^ | 29.2 ± 0.9^c^ | 2.28 ± 0.17^ab^ | 0.96 ± 0.06^ab^ | 2.49 ± 0.09^a^ | 3.6 ± 0.3^ab^ | 3.08 ± 0.25^a^ | 0.28 ± 0.04^a^ | |
| Sna7 | 5.5 | 37 | 25 | 5 | 101 ± 5^a^ | 2.75 ± 0.11^a^ | 29.48 ± 0.07^a^ | 2.47 ± 0.03^a^ | 1.04 ± 0.04^a^ | 2.79 ± 0.12^a^ | 4.0 ± 0.5^a^ | 3.34 ± 0.09^a^ | 0.299 ± 0.013^a^ | |
| Sna8 | 7.0 | 37 | 25 | 5 | 92.9 ± 2.7^ab^ | 1.10 ± 0.03^d^ | 4.2 ± 0.6^f^ | 1.73 ± 0.13^c^ | 0.899 ± 0.020^b^ | 1.70 ± 0.12^bc^ | 2.77 ± 0.29^b^ | 1.94 ± 0.06^d^ | 0.163 ± 0.007^d^ | |
| Mean values with different letters (a, b, etc.) within the same column are statistically different (*p < 0.05*)  DHC: dihydrochalcones | | | | | | | | | | | | | |  |

Table S3 Summarized aglycone yields by hydrolysis with cellobiase (5 U or 50 Units/mL) at various parameters for the methanolic flower extracts (n = 3 ± SD).

| **Parameters** | | | | ***B. ferulifolia*** | | | ***C. grandiflora*** | | | ***P. × hybrida*** | | ***T. pratense*** | | |
| --- | --- | --- | --- | --- | --- | --- | --- | --- | --- | --- | --- | --- | --- | --- |
| No. | pH | T  (°C) | t  (min) | Σ(aurones)  (µg/g) | Σ(chalcones)  (mg/g) | Σ(flavones)  (µg/g) | Σ(aurones)  (mg/g) | Σ(chalcones)  (mg/g) | Σ(flavones)  (mg/g) | Σ(DHF)  (mg/g) | Σ(flavonols)  (mg/g) | Σ(flavonols)  (mg/g) | Σ(isoflavones)  (mg/g) | |
| Cbi1 | 3.5 | 37 | 25 | 25.9 ± 2.0^a^ | 1.67 ± 0.16^a^ | 78 ± 6^a^ | 2.2 ± 0.3^a^ | 14.5 ± 1.2^a^ | 1.30 ± 0.05^a^ | 0.809 ± 0.014^a^ | 0.107 ± 0.004^c^ | 0.34 ± 0.05^a^ | 0.108 ± 0.006^ab^ | |
| Cbi2 | 4.0 | 37 | 25 | 24.2 ± 1.6^a^ | 1.61 ± 0.07^a^ | 74.4 ± 2.9^a^ | 2.2 ± 0.3^a^ | 14.8 ± 1.3^a^ | 1.38 ± 0.09^a^ | 0.79 ± 0.03^a^ | 0.124 ± 0.003^b^ | 0.37 ± 0.03^a^ | 0.111 ± 0.011^a^ | |
| Cbi3 | 4.5 | 37 | 25 | 23.9 ± 2.1^a^ | 1.68 ± 0.05^a^ | 78 ± 4^a^ | 2.1 ± 0.4^a^ | 14.3 ± 0.4^a^ | 1.35 ± 0.11^a^ | 0.821 ± 0.020^a^ | 0.1388 ± 0.0029^a^ | 0.39 ± 0.04^a^ | 0.111 ± 0.011^a^ | |
| Cbi4 | 4.0 | 25 | 25 | 25.4 ± 1.9^a^ | 1.60 ± 0.06^a^ | 69.0 ± 2.0^b^ | 2.03 ± 0.21^a^ | 13.6 ± 1.9^a^ | 1.26 ± 0.11^a^ | 0.788 ± 0.007^a^ | 0.077 ± 0.005^d^ | 0.24 ± 0.03^a^ | 0.085 ± 0.007^bc^ | |
| Cbi5 | 4.5 | 37 | 10 | 23.1 ± 0.9^a^ | 1.59 ± 0.11^a^ | 62 ± 4^ab^ | 2.40 ± 0.28^a^ | 15.4 ± 1.2^a^ | 1.34 ± 0.05^a^ | 0.795 ± 0.018^a^ | 0.0732 ± 0.0025^d^ | 0.23 ± 0.03^a^ | 0.081 ± 0.006^c^ | |
| Mean values with different letters (a, b, etc.) within the same column are statistically different (*p < 0.05*)  DHF: dihydroflavonols | | | | | | | | | | | | | |  |

Table S4 Summarized aglycone yields by hydrolysis with cellobiase (5 U or 50 Units/mL) at various parameters for the methanolic leaf extracts (n = 3 ± SD).

| **Parameters** | | | | ***F. sylvatica*** | | ***M. × domestica*** | | ***M. × piperita*** | | ***Q. robur*** | ***R. pseudoacacia*** | | |
| --- | --- | --- | --- | --- | --- | --- | --- | --- | --- | --- | --- | --- | --- |
| No. | pH | T  (°C) | t  (min) | Σ(flavones)  (µg/g) | Σ(flavonols)  (mg/g) | Σ(DHC)  (mg/g) | Σ(flavonols)  (mg/g) | Σ(flavones)  (mg/g) | Σ(flavanones)  (mg/g) | Σ(flavonols)  (mg/g) | Σ(flavones)  (mg/g) | Σ(flavonols)  (mg/g) | |
| Cbi1 | 3.5 | 37 | 25 | 70.4 ± 1.5^b^ | 0.0805 ± 0.0018^bc^ | 21.4 ± 0.4^b^ | 1.27 ± 0.07^bc^ | 0.731 ± 0.016^a^ | 2.67 ± 0.12^a^ | 1.77 ± 0.11^c^ | 1.53 ± 0.09^cd^ | 9.2 ± 0.6^c^ | |
| Cbi2 | 4.0 | 37 | 25 | 72 ± 6^b^ | 0.094 ± 0.007^b^ | 26.6 ± 2.2^a^ | 1.44 ± 0.08^a^ | 0.76 ± 0.06^a^ | 2.67 ± 0.25^a^ | 2.07 ± 0.13^b^ | 1.582 ± 0.022^bc^ | 13.1 ± 0.8^b^ | |
| Cbi3 | 4.5 | 37 | 25 | 87 ± 8^a^ | 0.125 ± 0.009^a^ | 27.2 ± 0.5^a^ | 1.503 ± 0.025^a^ | 0.83 ± 0.05^a^ | 2.72 ± 0.17^a^ | 2.51 ± 0.07^a^ | 2.07 ± 0.03^a^ | 16.1 ± 0.8^a^ | |
| Cbi4 | 4.0 | 25 | 25 | 72 ± 4^ab^ | 0.0683 ± 0.0017^c^ | 26.4 ± 1.5^a^ | 1.37 ± 0.08^ab^ | 0.73 ± 0.11^a^ | 2.63 ± 0.25^a^ | 1.50 ± 0.08^d^ | 1.39 ± 0.05^d^ | 7.4 ± 0.6^c^ | |
| Cbi5 | 4.5 | 37 | 10 | 55 ± 6^c^ | 0.071 ± 0.006^c^ | 25.9 ± 1.0^a^ | 1.1645 ± 0.0011^c^ | 0.80 ± 0.05^a^ | 2.69 ± 0.21^a^ | 1.51 ± 0.04^d^ | 1.73 ± 0.09^b^ | 7.8 ± 1.1^c^ | |
| Mean values with different letters (a, b, etc.) within the same column are statistically different (*p < 0.05*)  DHC: dihydrochalcones | | | | | | | | | | | | |  |

Table S5 Summarized aglycone yields by hydrolysis with cellulase (20 U or 100 Units/mL) at various parameters for the methanolic flower extracts (n = 3 ± SD).

| **Parameters** | | | | ***B. ferulifolia*** | | | ***C. grandiflora*** | | | ***P. × hybrida*** | | ***T. pratense*** | | |
| --- | --- | --- | --- | --- | --- | --- | --- | --- | --- | --- | --- | --- | --- | --- |
| No. | pH | T  (°C) | t  (min) | Σ(aurones)  (µg/g) | Σ(chalcones)  (mg/g) | Σ(flavones)  (µg/g) | Σ(aurones)  (mg/g) | Σ(chalcones)  (mg/g) | Σ(flavones)  (mg/g) | Σ(DHF)/  (mg/g) | Σ(flavonols)  (µg/g) | Σ(flavonols)  (µg/g) | Σ(isoflavones)  (µg/g) | |
| Cel1 | 5.5 | 52 | 25 | 20.8 ± 2.2^a^ | 1.38 ± 0.08^a^ | 60 ± 4^a^ | 2.2 ± 0.4^a^ | 13.5 ± 0.6^a^ | 1.09 ± 0.07^a^ | 0.696 ± 0.026^a^ | 6.5 ± 0.4^a^ | 45 ± 3^a^ | 45 ± 5^a^ | |
| Cel2 | 6.0 | 52 | 25 | 20.3 ± 1.4^ab^ | 1.28 ± 0.04^ab^ | 61.4 ± 2.7^a^ | 2.4 ± 0.5^a^ | 14.1 ± 0.9^a^ | 1.15 ± 0.16^a^ | 0.79 ± 0.04^a^ | 5.8 ± 0.6^ab^ | 23.5 ± 2.8^b^ | 34 ± 4^b^ | |
| Cel3 | 6.5 | 52 | 25 | 16.4 ± 0.7^b^ | 1.11 ± 0.11^b^ | 53 ± 6^a^ | 1.8 ± 0.3^a^ | 8.6 ± 0.6^b^ | 0.79 ± 0.04^b^ | 0.714 ± 0.006^a^ | 5.7 ± 0.5^b^ | 14.8 ± 1.2^c^ | 40 ± 3^ab^ | |
| Cel4 | 6.0 | 25 | 25 | 21.0 ± 2.2^a^ | 1.2723 ± 0.0021^ab^ | 57 ± 4^a^ | 2.43 ± 0.28^a^ | 14.1 ± 1.6^a^ | 1.18 ± 0.06^a^ | 0.74 ± 0.05^a^ | 4.7 ± 0.4^b^ | 4.1 ± 0.3^d^ | 35 ± 3^b^ | |
| Cel5 | 6.0 | 52 | 10 | 19.6 ± 1.0^ab^ | 1.30 ± 0.11^ab^ | 61 ± 5^a^ | 2.4 ± 0.4^a^ | 14.6 ± 1.2^a^ | 1.31 ± 0.05^a^ | 0.78 ± 0.05^a^ | 6.5 ± 0.9^a^ | 14.8 ± 1.2^c^ | 36.7 ± 2.0^ab^ | |
| Mean values with different letters (a, b, etc.) within the same column are statistically different (*p < 0.05*)  DHF: dihydroflavonols | | | | | | | | | | | | | |  |

Table S6 Summarized aglycone yields by hydrolysis with cellulase (20 U or 100 Units/mL) at various parameters for the methanolic leaf extracts (n = 3 ± SD).

| **Parameters** | | | | ***F. sylvatica*** | | ***M. × domestica*** | | ***M. × piperita*** | | ***Q. robur*** | ***R. pseudoacacia*** | | |
| --- | --- | --- | --- | --- | --- | --- | --- | --- | --- | --- | --- | --- | --- |
| No. | pH | T  (°C) | t  (min) | Σ(flavones)  (µg/g) | Σ(flavonols)  (µg/g) | Σ(DHC)  (mg/g) | Σ(flavonols)  (mg/g) | Σ(flavones)  (mg/g) | Σ(flavanones)  (mg/g) | Σ(flavonols)  (mg/g) | Σ(flavones)  (µg/g) | Σ(flavonols)  (µg/g) | |
| Cel1 | 5.5 | 52 | 25 | 79 ± 6^ab^ | 59.0 ± 1.3^a^ | 6.2 ± 0.4^a^ | 0.43 ± 0.04^a^ | n.d. | n.d. | 0.42 ± 0.03^a^ | 85 ± 7^a^ | 30.1 ± 2.6^a^ | |
| Cel2 | 6.0 | 52 | 25 | 75.8 ± 2.2^b^ | 21.8 ± 0.6^b^ | 3.29 ± 0.11^b^ | 0.308 ± 0.020^b^ | n.d. | n.d. | 0.291 ± 0.011^b^ | 78 ± 7^a^ | 22.0 ± 1.5^b^ | |
| Cel3 | 6.5 | 52 | 25 | 79 ± 5^ab^ | 11.1 ± 0.9^c^ | 1.101 ± 0.020^d^ | 0.142 ± 0.004^d^ | n.d. | n.d. | 0.01879 ± 0.00005^e^ | 73 ± 7^a^ | 11.3 ± 1.4^b^ | |
| Cel4 | 6.0 | 25 | 25 | 85.3 ± 2.5^ab^ | n.d. | 1.55 ± 0.08^d^ | 0.195 ± 0.014^cd^ | n.d. | n.d. | 0.134 ± 0.005^c^ | 80 ± 6^a^ | n.d. | |
| Cel5 | 6.0 | 52 | 10 | 88.5 ± 1.1^a^ | 19.7 ± 2.3^b^ | 2.5 ± 0.4^c^ | 0.27 ± 0.05^bc^ | n.d. | n.d. | 0.064 ± 0.012^d^ | 74.6 ± 1.9^a^ | 18 ± 3^c^ | |
| Mean values with different letters (a, b, etc.) within the same column are statistically different (*p < 0.05*)  DHC: dihydrochalcones  n.d. not detected | | | | | | | | | | | | |  |

Table S7 Summarized aglycone yields by hydrolysis with β-glucosidase (20 U or 100 Units/mL) at various parameters for the methanolic flower extracts (n = 3 ± SD).

| **Parameters** | | | | ***B. ferulifolia*** | | | ***C. grandiflora*** | | | ***P. × hybrida*** | | ***T. pratense*** | | |
| --- | --- | --- | --- | --- | --- | --- | --- | --- | --- | --- | --- | --- | --- | --- |
| No. | pH | T  (°C) | t  (min) | Σ(aurones)  (µg/g) | Σ(chalcones)  (mg/g) | Σ(flavones)  (µg/g) | Σ(aurones)  (mg/g) | Σ(chalcones)  (mg/g) | Σ(flavones)  (mg/g) | Σ(DHF)  (mg/g) | Σ(flavonols)  (µg/g) | Σ(flavonols)  (µg/g) | Σ(isoflavones)  (µg/g) | |
| Glu1 | 4.5 | 37 | 25 | 21.1 ± 1.1^a^ | 1.25 ± 0.05^a^ | 78 ± 4^a^ | 2.30 ± 0.22^a^ | 14.6 ± 1.6^a^ | 1.21 ± 0.03^a^ | 0.722 ± 0.021^a^ | 28.0 ± 1.2^a^ | 4.97 ± 0.21^ab^ | 57 ± 5^a^ | |
| Glu2 | 5.0 | 37 | 25 | 18.6 ± 0.5^a^ | 1.09 ± 0.11^a^ | 64 ± 7^b^ | 2.3 ± 0.3^a^ | 15.0 ± 1.2^a^ | 1.25 ± 0.05^a^ | 0.65 ± 0.07^a^ | 27 ± 3^a^ | 5.6 ± 0.7^a^ | 47 ± 6^ab^ | |
| Glu3 | 5.5 | 37 | 25 | 20.1 ± 2.2^a^ | 1.27 ± 0.08^a^ | 66 ± 3^b^ | 2.4 ±0.6^a^ | 15.0 ± 1.0^a^ | 1.30 ± 0.21^a^ | 0.70 ± 0.04^a^ | 28.9 ± 2.3^a^ | 5.4 ± 0.6^a^ | 38 ± 5^bc^ | |
| Glu4 | 5.0 | 25 | 25 | 20.8 ± 0.9^a^ | 1.24 ± 0.07^a^ | 63.7 ± 0.9^b^ | 2.4 ± 0.4^s^ | 15.6 ± 0.6^a^ | 1.27 ± 0.11^a^ | 0.71 ± 0.05^a^ | 27.8 ± 2.6^a^ | 2.57 ± 0.24^c^ | 34.4 ± 2.2^c^ | |
| Glu5 | 5.5 | 37 | 10 | 18.5 ± 0.8^a^ | 1.263 ± 0.009^a^ | 64 ± 3^b^ | 2.27 ± 0.22^a^ | 14.6 ± 1.9^a^ | 1.20 ± 0.08^a^ | 0.714 ± 0.024^a^ | 29.9 ± 1.8^a^ | 4.0 ± 0.4^b^ | 34.0 ± 2.3^c^ | |
| Mean values with different letters (a, b, etc.) within the same column are statistically different (*p < 0.05*)  DHF: dihydroflavonols | | | | | | | | | | | | | |  |

Table S8 Summarized aglycone yields by hydrolysis with β-glucosidase (20 U or 100 Units/mL) at various parameters for the methanolic leaf extracts (n = 3 ± SD).

| **Parameters** | | | | | ***F. sylvatica*** | | | ***M. × domestica*** | | | ***M. × piperita*** | | | ***Q. robur*** | | ***R. pseudoacacia*** | | | |
| --- | --- | --- | --- | --- | --- | --- | --- | --- | --- | --- | --- | --- | --- | --- | --- | --- | --- | --- | --- |
| No. | pH | T  (°C) | t  (min) | Σ(flavones)  (µg/g) | | Σ(flavonols)  (µg/g) | Σ(DHC)  (mg/g) | | Σ(flavonols)  (mg/g) | Σ(flavones)  (µg/g) | | Σ(flavanones)  (µg/g) | Σ(flavonols)  (mg/g) | | Σ(flavones)  (mg/g) | | Σ(flavonols)  (µg/g) | |  |
| Glu1 | 4.5 | 37 | 25 | 72.3 ± 0.5^a^ | | 55.1 ± 0.7^b^ | 14.0 ± 0.4^b^ | | 0.177 ± 0.005^a^ | 26 ± 5^cd^ | | 14.4 ± 0.5^b^ | 1.41 ± 0.14^a^ | | 0.0898 ± 0.0024^a^ | | 69 ± 3^a^ | |  |
| Glu2 | 5.0 | 37 | 25 | 78 ± 5^a^ | | 71 ± 5^a^ | 15.1 ± 0.4^a^ | | 0.184 ± 0.009^a^ | 41 ± 5^b^ | | 17.4 ± 0.6^ab^ | 1.50 ± 0.11^a^ | | 0.093 ± 0.008^a^ | | 70 ± 6^a^ | |  |
| Glu3 | 5.5 | 37 | 25 | 75 ± 12^a^ | | 67 ± 9^ab^ | 10.3 ± 0.5^c^ | | 0.167 ± 0.006^ab^ | 54 ± 3^a^ | | 21 ± 4^a^ | 1.53 ± 0.08^a^ | | 0.102 ± 0.010^a^ | | 72 ± 7^a^ | |  |
| Glu4 | 5.0 | 25 | 25 | 78.8 ± 2.3^a^ | | 55.7 ± 1.8^b^ | 9.36 ± 0.20^c^ | | 0.154 ± 0.007^b^ | 20 ± 4^d^ | | 14.5 ± 0.7^b^ | 1.54 ± 0.18^a^ | | 0.085 ± 0.010^a^ | | 67 ± 7^a^ | |  |
| Glu5 | 5.5 | 37 | 10 | 77 ± 4^a^ | | 59 ± 4^b^ | 7.56 ± 0.18^d^ | | 0.154 ± 0.008^b^ | 36 ± 4^bc^ | | n.d. | 1.47 ± 0.13^a^ | | 0.093 ± 0.008^a^ | | 70 ± 4^a^ | |  |
| Mean values with different letters (a, b, etc.) within the same column are statistically different (*p < 0.05*)  DHC: dihydrochalcones  n.d. not detected | | | | | | | | | | | | | | | | | |  |  |

Table S9 Summarized aglycone yields by hydrolysis with pectinase (20 U or 100 Units/mL) at various parameters for the methanolic flower extracts (n = 3 ± SD).

| **Parameters** | | | | ***B. ferulifolia*** | | | ***C. grandiflora*** | | | ***P. × hybrida*** | | ***T. pratense*** | | |
| --- | --- | --- | --- | --- | --- | --- | --- | --- | --- | --- | --- | --- | --- | --- |
| No. | pH | T  (°C) | t  (min) | Σ(aurones)  (µg/g) | Σ(chalcones)  (mg/g) | Σ(flavones)  (µg/g) | Σ(aurones)  (mg/g) | Σ(chalcones)  (mg/g) | Σ(flavones)  (mg/g) | Σ(DHF)  (mg/g) | Σ(flavonols)  (µg/g) | Σ(flavonols)  (mg/g) | Σ(isoflavones)  (mg/g) | |
| Pec1 | 3.5 | 40 | 25 | 17.1 ± 2.6^a^ | 0.62 ± 0.07^c^ | 30 ± 4^c^ | 0.61 ± 0.12^c^ | 2.26 ± 0.20^c^ | 0.41 ± 0.03^c^ | 0.35 ± 0.03^c^ | 22 ± 4^bc^ | 0.48 ± 0.06^a^ | 45 ± 3^a^ | |
| Pec2 | 4.0 | 40 | 25 | 21.6 ± 1.9^a^ | 1.20 ± 0.13^ab^ | 54 ± 6^a^ | 1.57 ± 0.21^ab^ | 5.0 ± 0.8^b^ | 1.17 ± 0.12^a^ | 0.72 ± 0.06^ab^ | 36 ± 7^ab^ | 0.50 ± 0.06^a^ | 51.3 ± 2.3^a^ | |
| Pec3 | 4.5 | 40 | 25 | 21.9 ± 0.9^a^ | 1.29 ± 0.06^ab^ | 60 ± 4^a^ | 1.9 ± 0.4^a^ | 7.3 ± 0.4^a^ | 1.36 ± 0.11^a^ | 0.75 ± 0.07^a^ | 47 ± 9^a^ | 0.46 ± 0.04^a^ | 47 ± 7^a^ | |
| Pec4 | 4.0 | 25 | 25 | 19.5 ± 2.4^a^ | 1.03 ± 0.16^b^ | 47 ± 8^a^ | 1.07 ± 0.18^bc^ | 3.5 ± 0.5^c^ | 0.72 ± 0.09^b^ | 0.60 ± 0.03^b^ | 28 ± 4^bc^ | 0.391 ± 0.023^a^ | 43 ± 5^a^ | |
| Pec5 | 4.5 | 40 | 10 | 19.6 ± 1.1^a^ | 1.36 ± 0.08^a^ | 55.5 ± 1.2^a^ | 1.7 ± 0.3^ab^ | 6.30 ± 0.21^ab^ | 1.20 ± 0.13^a^ | 0.746 ± 0.014^a^ | 20.6 ± 0.8^c^ | 0.46 ± 0.07^a^ | 42 ± 4^a^ | |
| Mean values with different letters (a, b, etc.) within the same column are statistically different (*p < 0.05*)  DHF: dihydroflavonols | | | | | | | | | | | | | |  |

Table S10 Summarized aglycone yields by hydrolysis with pectinase (20 U or 100 Units/mL) at various parameters for the methanolic leaf extracts (n = 3 ± SD).

| **Parameters** | | | | ***F. sylvatica*** | | ***M. × domestica*** | | ***M. × piperita*** | | ***Q. robur*** | ***R. pseudoacacia*** | |
| --- | --- | --- | --- | --- | --- | --- | --- | --- | --- | --- | --- | --- |
| No. | pH | T  (°C) | t  (min) | Σ(flavones)  (µg/g) | Σ(flavonols)  (mg/g) | Σ(DHC)  (mg/g) | Σ(flavonols)  (mg/g) | Σ(flavones)  (mg/g) | Σ(flavanones)  (mg/g) | Σ(flavonols)  (mg/g) | Σ(flavones)  (mg/g) | Σ(flavonols)  (mg/g) |
| Pec1 | 3.5 | 40 | 25 | 22.2± 0.7^c^ | 0.1090 ± 0.0029^b^ | 1.85 ± 0.18^ab^ | 0.93 ± 0.05^a^ | 0.36 ± 0.04^b^ | 0.68 ± 0.12^c^ | 0.87 ± 0.10^b^ | 0.405 ± 0.011^d^ | 0.103 ± 0.007^b^ |
| Pec2 | 4.0 | 40 | 25 | 64 ± 7^a^ | 0.123 ± 0.007^a^ | 2.19 ± 0.04^ab^ | 1.04 ± 0.13^a^ | 0.80 ± 0.04^a^ | 2.22 ± 0.25^b^ | 1.07 ± 0.04^a^ | 1.15 ± 0.07^b^ | 0.134 ± 0.011^a^ |
| Pec3 | 4.5 | 40 | 25 | 77 ± 5^a^ | 0.124 ± 0.009^a^ | 2.5 ± 0.3^a^ | 0.97 ± 0.06^a^ | 0.89 ± 0.05^a^ | 2.78 ± 0.22^a^ | 1.07 ± 0.04^a^ | 1.30 ± 0.03^a^ | 0.143 ± 0.016^a^ |
| Pec4 | 4.0 | 25 | 25 | 47 ± 5^b^ | 0.102 ± 0.003^b^ | 1.72 ± 0.08^b^ | 0.83 ± 0.04^a^ | 0.39 ± 0.08^b^ | 0.93 ± 0.07^c^ | 0.99 ± 0.09^ab^ | 0.84 ± 0.05^c^ | 0.118 ± 0.006^ab^ |
| Pec5 | 4.5 | 40 | 10 | 72 ± 9^a^ | 0.0966 ± 0.0004^b^ | 2.4 ± 0.4^ab^ | 0.90 ± 0.08^a^ | 0.85 ± 0.04^a^ | 2.61 ± 0.14^ab^ | 0.99 ± 0.04^ab^ | 1.19 ± 0.08^ab^ | 0.115 ± 0.014^ab^ |
| Mean values with different letters (a, b, etc.) within the same column are statistically different (*p < 0.05*)  DHC: dihydrochalcones | | | | | | | | | | | | |

Table S11 Summarized aglycone yields by acidic hydrolysis at various parameters for the methanolic flower extracts (n = 3 ± SD).

| **Parameters** | | | | ***B. ferulifolia*** | | | ***C. grandiflora*** | | | ***P. × hybrida*** | | ***T. pratense*** | |  |
| --- | --- | --- | --- | --- | --- | --- | --- | --- | --- | --- | --- | --- | --- | --- |
| No. | c_HCl_ (M) | T  (°C) | t  (min) | Σ(aurones)  (µg/g) | Σ(chalcones)  (mg/g) | Σ(flavones)  (µg/g) | Σ(aurones)  (mg/g) | Σ(chalcones)  (mg/g) | Σ(flavones)  (mg/g) | Σ(DHF)  (mg/g) | Σ(flavonols)  (mg/g) | Σ(flavonols)  (mg/g) | Σ(isoflavones)  (mg/g) | |
| HCl1 | 1 | 40 | 60 | n.d. | 0.07891 ± 0.00026^c^ | 9.57 ± 0.09^d^ | 0.051 ± 0.008^c^ | 0.212 ± 0.012^d^ | n.d. | 0.01307 ± 0.00009^d^ | 0.0133 ± 0.0004^f^ | 0.227 ± 0.017^c^ | 0.0132 ± 0.0007^c^ | |
| HCl2 | 2 | 40 | 60 | n.d. | 0.1086 ± 0.0007^c^ | 10.44 ± 0.05^d^ | 0.144 ± 0.019^c^ | 0.57 ± 0.04^d^ | 0.0175 ± 0.0013^f^ | 0.0203 ± 0.0006^f^ | 0.0313 ± 0.0012^e^ | 0.76 ± 0.08^c^ | 0.0220 ± 0.0023^bc^ | |
| HCl3 | 1 | 70 | 60 | n.d. | 0.236 ± 0.008^b^ | 13.1 ± 0.5^d^ | 0.71 ± 0.09^c^ | 2.07 ± 0.20^c^ | 0.133 ± 0.007^e^ | 0.0610 ± 0.0008^e^ | 0.0556 ± 0.0009^d^ | 2.94 ± 0.29^a^ | 0.0245 ± 0.0020^bc^ | |
| HCl4 | 2 | 70 | 60 | 16.8 ± 1.6^b^ | 0.279 ± 0.013^a^ | 23.5 ± 1.6^c^ | 1.42 ± 0.26^b^ | 2.61 ± 0.11^b^ | 0.336 ± 0.022^d^ | 0.139 ± 0.004^f^ | 0.0898 ± 0.0025^c^ | 3.0 ± 0.3^a^ | 0.0352 ± 0.0023^b^ | |
| HCl5 | 1 | 100 | 60 | 26.8 ± 1.5^a^ | 0.291 ± 0.021^a^ | 70 ± 5^ab^ | 2.9 ± 0.4^a^ | 3.3 ± 0.4^a^ | 1.36 ± 0.05^a^ | 0.438 ± 0.003^a^ | 0.159 ± 0.004^b^ | 2.70 ± 0.29^ab^ | 0.1277 ± 0.014^a^ | |
| HCl6 | 2 | 100 | 60 | 25.6 ± 1.5^a^ | 0.296 ± 0.021^a^ | 73 ± 4^a^ | 2.5 ± 0.4^a^ | 2.81 ± 0.13^ab^ | 1.24 ± 0.05^b^ | 0.4038 ± 0.0015^b^ | 0.1610 ± 0.0026^b^ | 2.3 ± 0.4^ab^ | 0.1361 ± 0.0012^a^ | |
| HCl7 | 1 | 100 | 120 | 24.7 ± 1.3^a^ | 0.284 ± 0.020^a^ | 64 ± 3^b^ | 2.5 ± 0.3^a^ | 3.00 ± 0.26^ab^ | 1.03 ± 0.05^c^ | 0.376 ± 0.007^c^ | 0.1725 ± 0.0020^a^ | 2.1 ± 0.4^b^ | 0.121 ± 0.008^a^ | |
| Mean values with different letters (a, b, etc.) within the same column are statistically different (*p < 0.05*)  DHF: dihydroflavonols  n.d. not detected | | | | | | | | | | | | | | |

Table S12 Summarized aglycone yields by acidic hydrolysis at various parameters for the methanolic leaf extracts (n = 3 ± SD).

| **Parameters** | | | | ***F. sylvatica*** | | ***M. × domestica*** | | ***M. × piperita*** | | ***Q. robur*** | ***R. pseudoacacia*** | |
| --- | --- | --- | --- | --- | --- | --- | --- | --- | --- | --- | --- | --- |
| No. | c_HCl_ (M) | T  (°C) | t  (min) | Σ(flavones)  (µg/g) | Σ(flavonols)  (mg/g) | Σ(DHC)  (mg/g) | Σ(flavonols)  (mg/g) | Σ(flavones)  (mg/g) | Σ(flavanones)  (mg/g) | Σ(flavonols)  (mg/g) | Σ(flavones)  (mg/g) | Σ(flavonols)  (mg/g) |
| HCl1 | 1 | 40 | 60 | n.d. | 0.518 ± 0.019^d^ | 1.90 ± 0.04^d^ | 1.04 ± 0.04^d^ | 0.0155 ± 0.0011^d^ | 0.0089 ± 0.0015^d^ | 0.53 ± 0.05^d^ | 0.0156 ± 0.0011^d^ | 0.027 ± 0.004^c^ |
| HCl2 | 2 | 40 | 60 | n.d. | 1.28 ± 0.16^c^ | 4.81 ± 0.15^d^ | 1.61 ± 0.06^c^ | 0.024 ± 0.005^d^ | 0.0196 ± 0.0027^d^ | 1.08 ± 0.06^c^ | 0.0218 ± 0.0014^d^ | 0.0303 ± 0.0023^c^ |
| HCl3 | 1 | 70 | 60 | 5.3 ± 0.4^c^ | 2.02 ± 0.13^a^ | 17.5 ± 0.3^c^ | 2.13 ± 0.13^b^ | 0.0670 ± 0.0020^cd^ | 0.173 ± 0.013^cd^ | 2.20 ± 0.18^a^ | 0.102 ± 0.006^d^ | 0.112 ± 0.007^ab^ |
| HCl4 | 2 | 70 | 60 | 12.8 ± 0.4^b^ | 1.98 ± 0.12^a^ | 21.6 ± 0.7^bc^ | 2.34 ± 0.10^ab^ | 0.146 ± 0.006^c^ | 0.46 ± 0.03^c^ | 2.41 ± 0.15^a^ | 0.262 ± 0.025^c^ | 0.127± 0.010^a^ |
| HCl5 | 1 | 100 | 60 | 69.8 ± 2.1^a^ | 1.11 ± 0.07^c^ | 27.9 ± 1.6^a^ | 2.50 ± 0.08^a^ | 0.855 ± 0.08^a^ | 2.40 ± 0.26^a^ | 2.07 ± 0.14^ab^ | 0.92 ± 0.08^a^ | 0.125 ± 0.012^a^ |
| HCl6 | 2 | 100 | 60 | 67.4 ± 0.9^a^ | 1.085 ± 0.020^c^ | 26.3 ± 0.6^a^ | 2.34 ± 0.06^ab^ | 0.801 ± 0.019^a^ | 2.02 ± 0.10^ab^ | 1.71 ± 0.12^b^ | 1.02 ± 0.06^a^ | 0.127 ± 0.010^a^ |
| HCl7^1^ | 1 | 100 | 120 | 10.5 ± 0.6^b^ | 1.69 ± 0.04^b^ | 22 ± 3^b^ | 2.15 ± 0.04^b^ | 0.69 ± 0.04^b^ | 2.00 ± 0.24^b^ | 2.41 ± 0.20^a^ | 0.72 ± 0.04^b^ | 0.094 ± 0.006^b^ |
| Mean values with different letters (a, b, etc.) within the same column are statistically different (*p < 0.05*)  DHC: dihydrochalcones  ^1^*F. sylvatica* and *Q. robur* were performed at 70 °C | | | | | | | | | | | | |

Table S13 Aglycone yields from flower extracts of B. ferulifolia. Enzymatic hydrolysis (25 min): Sna7: 5 mg Snailase (pH 5.5, 37 °C); Cbi3: 5 U Cellobiase (pH 4.5, 37 °C); Cel1: 20 U Cellulase (pH 5.5, 52 °C); Pec3: 20 U Pectinase (pH 4.5, 40 °C); Glu1: 20 U β-Glucosidase (pH 4.5, 37 °C). Acidic hydrolysis (60 min): HCl5: 1M HCl at 100 °C. (n = 3 ± SD).

| **Exp.** | **Okanin**  **(mg/g)** | **Luteolin**  **(mg/g)** | **Lanceoletin**  **(µg/g)** | **Butein**  **(µg/g)** | **Maritimetin**  **(µg/g)** |
| --- | --- | --- | --- | --- | --- |
| Sna7 | 1.78 ± 0.09^a^ | 0.116 ± 0.006^a^ | 57 ± 3^a^ | 32.1 ± 1.4^a^ | 28.8 ± 2.2^a^ |
| Cbi3 | 1.60 ± 0.05^b^ | 0.078 ± 0.004^b^ | 43.8 ± 1.5^b^ | 34.4 ± 1.4^a^ | 23.9 ± 2.1^bc^ |
| Cel1 | 1.32 ± 0.08^c^ | 0.060 ± 0.004^c^ | 37.2 ± 1.2^b^ | 22.9 ± 2.6^b^ | 20.8 ± 2.2^c^ |
| Glu1 | 1.19 ± 0.05^c^ | 0.078 ± 0.003^b^ | 36.2 ± 1.8^b^ | 22.2 ± 0.7^b^ | 21.1 ± 1.1^c^ |
| Pec3 | 1.26 ± 0.06^c^ | 0.060 ± 0.004^c^ | 15.6 ± 1.4^c^ | 22.5 ± 1.0^b^ | 21.9 ± 0.9^c^ |
| HCl5 | 0.28 ± 0.02^d^ | 0.070 ± 0.005^bc^ | 9.6 ± 0.9^c^ | 4.85 ± 0.19^c^ | 26.8 ± 1.5^ab^ |
| Mean values with different letters (a, b, etc.) within the same column are statistically different (*p < 0.05*). Lanceoletin and okanin calculated as butein equivalents; maritimetin as sulfuretin equivalents; luteolin as apigenin equivalents. | | | | | |

Table S14 Aglycone yields from flower extracts of C. grandiflora. Enzymatic hydrolysis (25 min): Sna7: 5 mg Snailase (pH 5.5, 37 °C); Glu3: 20 U β-Glucosidase (pH 5.5, 37 °C); Cbi3: 5 U Cellobiase (pH 4.5, 37 °C); Cel2: 20 U Cellulase (pH 6.0, 52 °C); Pec3: 20 U Pectinase (pH 4.5, 40 °C). Acidic hydrolysis (60 min): HCl5: 1M HCl at 100 °C. (n = 3 ± SD).

| **Exp.** | **Lanceoletin**  **(mg/g)** | **Okanin**  **(mg/g)** | **Leptosidin**  **(mg/g)** | **Luteolin**  **(mg/g)** | **Maritimetin**  **(mg/g)** | **Sulfuretin**  **(mg/g)** |
| --- | --- | --- | --- | --- | --- | --- |
| Sna7 | 12.4 ± 1.2^a^ | 3.4 ± 0.3^a^ | 2.14 ± 0.27^a^ | 1.55 ± 0.08^a^ | 0.25 ± 0.03^a^ | 0.104 ± 0.010^a^ |
| Cbi3 | 11.1 ± 0.3^ab^ | 3.16 ± 0.08^a^ | 1.8 ± 0.4^a^ | 1.35 ± 0.11^ab^ | 0.23 ± 0.04^a^ | 0.083 ± 0.018^a^ |
| Cel2 | 11.0 ± 0.7^b^ | 3.16 ± 0.18^a^ | 2.1 ± 0.5^a^ | 1.15 ± 0.16^b^ | 0.23 ± 0.04^a^ | 0.071 ± 0.017^a^ |
| Glu3 | 11.7 ± 0.8^ab^ | 3.30 ± 0.25^a^ | 2.1 ± 0.5^a^ | 1.30 ± 0.21^ab^ | 0.24 ± 0.06^a^ | 0.081 ± 0.016^a^ |
| Pec3 | 4.2 ± 0.3^c^ | 3.14 ± 0.05^a^ | 1.9 ± 0.3^a^ | 1.36 ± 0.11^ab^ | n.d.^*^ | 0.074 ± 0.010^a^ |
| HCl5 | 2.6 ± 0.3^c^ | 0.70 ± 0.07^b^ | 2.5 ± 0.3^a^ | 1.36 ± 0.05^ab^ | 0.28 ± 0.03^a^ | 0.102 ± 0.009^a^ |
| Mean values with different letters (a, b, etc.) within the same column are statistically different (*p < 0.05*). Lanceoletin and okanin calculated as butein equivalents; leptosidin and maritimetin as sulfuretin equivalents; luteolin as apigenin equivalents.  ^*^Not detectable, due to peak interferences | | | | | | |

Table S15 Aglycone yields from flower extracts of P. × hybrida. Enzymatic hydrolysis (25 min): Sna7: 5 mg Snailase (pH 5.5, 37 °C); Cbi3: 5 U Cellobiase (pH 4.5, 37 °C); Pec3: 20 U Pectinase (pH 4.5, 40 °C); Glu3: 20 U β-Glucosidase (pH 5.5, 37 °C); Cel2: 20 U Cellulase (pH 6.0, 52 °C). Acidic hydrolysis (60 min): HCl5: 1M HCl at 100 °C. (n = 3 ± SD).

| **Exp.** | **DHK**  **(mg/g)** | **Kaempferol**  **(mg/g)** |
| --- | --- | --- |
| Sna7 | 0.831 ± 0.028^a^ | 0.225 ± 0.007^a^ |
| Cbi3 | 0.821± 0.020^a^ | 0.1388 ± 0.0029^c^ |
| Cel2 | 0.79 ± 0.04^ab^ | 0.0058 ± 0.0006^f^ |
| Glu3 | 0.696 ± 0.04^b^ | 0.0289 ± 0.0023^e^ |
| Pec3 | 0.75 ± 0.07^ab^ | 0.047 ± 0.008^d^ |
| HCl5 | 0.438 ± 0.003^c^ | 0.159 ± 0.004^b^ |
| Mean values with different letters (a, b, etc.) within the same column are statistically different (p < 0.05). Kaempferol calculated as quercetin equivalents. | | |

Table S16 Aglycone yields from flower extracts of T. pratense. Enzymatic hydrolysis (25 min): Sna7: 5 mg Snailase (pH 5.5, 37 °C); Pec3: 20 U Pectinase (pH 4.5, 40 °C), Cbi3: 5 U Cellobiase (pH 4.5, 37 °C); Cel1: 20 U Cellulase (pH 5.5, 52 °C); Glu1: 20 U β-Glucosidase (pH 4.5, 37 °C). Acidic hydrolysis (60 min): HCl3: 1M HCl at 70 °C; HCl5: 1M HCl at 100 °C. (n = 3 ± SD).

| **Exp.** | **Kaempferol**  **(mg/g)** | **Quercetin**  **(mg/g)** | **Isorhamnetin**  **(mg/g)** | **Biochanin A**  **(µg/g)** | **Formononetin**  **(µg/g)** | **Genistein**  **(µg/g)** |
| --- | --- | --- | --- | --- | --- | --- |
| Sna7 | 1.34 ± 0.13^a^ | 1.29 ± 0.11^a^ | 0.225 ± 0.020^ab^ | 60 ± 7^ab^ | 36 ± 8^a^ | 25.4 ± 1.1^ab^ |
| Cbi3 | 0.39 ± 0.04^b^ | n.d. | n.d. | 50 ± 4^b^ | 36 ± 6^a^ | 25.3 ± 1.8^ab^ |
| Cel1 | 0.0143 ± 0.0010^c^ | 0.0307 ± 0.0022^b^ | n.d. | 19 ± 2^c^ | 8.0 ± 0.7^b^ | 18.2 ± 2.7^bc^ |
| Glu1 | n.d. | 0.00497 ± 0.00021^b^ | n.d. | 15.7 ± 1.1^c^ | 27 ± 3^a^ | 13.9 ± 2.7^c^ |
| Pec3 | 0.236 ± 0.022^bc^ | 0.189 ± 0.013^b^ | 0.038 ± 0.005^c^ | 13.9 ± 1.8^c^ | 8.8 ± 1.8^b^ | 24 ± 5^ab^ |
| HCl3 | 1.42 ± 0.11^a^ | 1.26 ± 0.11^a^ | 0.262 ± 0.021^a^ | 12.6 ± 0.9^c^ | 7.9 ± 0.4^b^ | 4.1 ± 0.7^d^ |
| HCl5 | 1.30 ± 0.15^a^ | 1.18 ± 0.11^a^ | 0.215 ± 0.024^b^ | 61 ± 5^a^ | 36 ± 8^a^ | 30.7 ± 1.9^a^ |
| Mean values with different letters (a, b, etc.) within the same column are statistically different (*p < 0.05*). Isorhamnetin and kaempferol calculated as quercetin equivalents; biochanin A and genistein as formononetin equivalents.  n.d. not detected | | | | | | |

Table S17 Aglycone yields from leaf extracts of M. × piperita. Enzymatic hydrolysis (25 min): Sna7: 5 mg Snailase (pH 5.5, 37 °C); Pec3: 20 U Pectinase (pH 4.5, 40 °C); Cbi3: 5 U Cellobiase (pH 4.5, 37 °C); Glu3: 20 U β-Glucosidase (pH 5.5, 37 °C); Cel1: 20 U Cellulase (pH 5.5, 52 °C). Acidic hydrolysis (60 min): HCl5: 1M HCl at 100 °C. (n = 3 ± SD).

| **Exp.** | **Eriodictyol /**  **(mg/g)** | **Luteolin /**  **(mg/g)** | **Hesperetin /**  **(mg/g)** | **Diosmetin /**  **(mg/g)** | **Apigenin /**  **(µg/g)** |
| --- | --- | --- | --- | --- | --- |
| Sna7 | 2.09 ± 0.11^a^ | 0.77 ± 0.06^a^ | 0.701 ± 0.019^a^ | 0.182 ± 0.024^a^ | 86 ± 6^a^ |
| Cbi3 | 2.03 ± 0.15^a^ | 0.58 ± 0.06^b^ | 0.683 ± 0.024^a^ | 0.166 ± 0.013^ab^ | 77 ± 5^ab^ |
| Cel1 | n.d. | n.d. | n.d. | n.d. | n.d. |
| Glu3 | 0.021 ± 0.004^b^ | 0.054 ± 0.003^c^ | n.d. | n.d. | n.d. |
| Pec3 | 2.09 ± 0.18^a^ | 0.63 ± 0.04^b^ | 0.69 ± 0.04^a^ | 0.182 ± 0.010^a^ | 74.7 ± 1.2^bc^ |
| HCl5 | 1.78 ± 0.20^a^ | 0.65 ± 0.07^ab^ | 0.61 ± 0.07^a^ | 0.140 ± 0.010^b^ | 65 ± 3^c^ |
| Mean values with different letters (a, b, etc.) within the same column are statistically different (*p < 0.05*). Hesperetin calculated as eriodictyol equivalents; diosmetin and luteolin as apigenin equivalents.  n.d. not detected | | | | | |

Table S18 Aglycone yields from leaf extracts of M. × domestica. Enzymatic hydrolysis (25 min): Sna7: 5 mg Snailase (pH 5.5, 37 °C); Cbi3: 5 U Cellobiase (pH 4.5, 37 °C); Glu2: 20 U β-Glucosidase (pH 5.0, 37 °C); Cel1: 20 U Cellulase (pH 5.5, 52 °C); Pec3: 20 U Pectinase (pH 4.5, 40 °C). Acidic hydrolysis (60 min): HCl5: 1M HCl at 100 °C. (n = 3 ± SD).

| **Exp.** | **Phloretin**  **(mg/g)** | **Quercetin**  **(mg/g)** | **Kaempferol**  **(mg/g)** | **Isorhamnetin**  **(mg/g)** |
| --- | --- | --- | --- | --- |
| Sna7 | 29.48 ± 0.07^a^ | 1.85 ± 0.04^a^ | 0.321 ± 0.006^a^ | 0.302 ± 0.012^a^ |
| Cbi3 | 27.2 ± 0.5^b^ | 1.233 ± 0.025^b^ | 0.188 ± 0.005^b^ | 0.081 ± 0.003^d^ |
| Cel1 | 6.2 ± 0.4^d^ | 0.38 ± 0.03^c^ | 0.048 ± 0.003^d^ | n.d. |
| Glu2 | 15.1 ± 0.4^c^ | 0.154 ± 0.007^d^ | 0.0301 ± 0.0015^d^ | n.d. |
| Pec3 | 2.5 ± 0.3^e^ | 0.72 ± 0.04^e^ | 0.111 ± 0.010^c^ | 0.139 ± 0.011^c^ |
| HCl5 | 27.9 ± 1.6^ab^ | 1.90 ± 0.06^a^ | 0.337 ± 0.010^a^ | 0.265 ± 0.011^b^ |
| Mean values with different letters (a, b, etc.) within the same column are statistically different (*p < 0.05*). Isorhamnetin and kaempferol calculated as quercetin equivalents.  n.d. not detected | | | | |

Table S19 Aglycone yields from leaf extracts of F. sylvatica. Enzymatic hydrolysis (25 min): Sna7: 5 mg Snailase (pH 5.5, 37 °C); Cbi3: 5 U Cellobiase (pH 4.5, 37 °C); Pec3: 20 U Pectinase (pH 4.5, 40 °C); Glu3: 20 U β-Glucosidase (pH 5.5, 37 °C); Cel1: 20 U Cellulase (pH 5.5, 52 °C). Acidic hydrolysis (60 min): HCl4: 2M HCl at 70 °C HCl5; 1M HCl at 100 °C. (n = 3 ± SD).

| **Exp.** | **Myricetin**  **(mg/g)** | **Quercetin**  **(mg/g)** | **Luteolin**  **(µg/g)** | **Kaempferol**  **(µg/g)** | **Apigenin**  **(mg/g)** |
| --- | --- | --- | --- | --- | --- |
| Sna7 | 2.01 ± 0.07^a^ | 0.69 ± 0.04^a^ | 64 ± 3^a^ | 56.1 ± 2.6^a^ | 36.9 ± 2.1^a^ |
| Cbi3 | 0.125 ± 0.009^d^ | n.d. | 58 ± 6^ab^ | n.d. | 28.1 ± 2.1^b^ |
| Cel1 | 0.0348 ± 0.0013^d^ | 0.0242 ± 0.0009^e^ | 59 ± 5^ab^ | n.d. | 23.1 ± 1.7^bc^ |
| Glu3 | 0.045 ± 0.007^d^ | 0.0222 ± 0.0027^e^ | 52 ± 8^ab^ | n.d. | 23 ± 0.4^c^ |
| Pec3 | 0.074 ± 0.005^d^ | 0.050 ± 0.004^d^ | 54 ± 4^ab^ | n.d. | 22.6 ± 1.5^bc^ |
| HCl4 | 1.45 ± 0.09^b^ | 0.496 ± 0.024^b^ | 12.8 ± 0.4^c^ | 36.4 ± 1.8^b^ | n.d. |
| HCl5 | 0.722 ± 0.06^c^ | 0.356 ± 0.012^c^ | 49.6 ± 1.5^b^ | 28.1 ± 1.1^c^ | 20.2 ± 0.5^c^ |
| Mean values with different letters (a, b, etc.) within the same column are statistically different (*p < 0.05*). Kaempferol and myricetin calculated as quercetin equivalents; luteolin as apigenin equivalents.  n.d. not detected | | | | | |

Table S20 Aglycone yields from leaf extracts of Q. robur. Enzymatic hydrolysis (25 min): Sna7: 5 mg Snailase (pH 5.5, 37 °C); Cbi3: 5 U Cellobiase (pH 4.5, 37 °C); Glu3: 20 U β-Glucosidase (pH 5.5, 37 °C); Pec3: 20 U Pectinase (pH 4.5, 40 °C); Cel1: 20 U Cellulase (pH 5.5, 52 °C). Acidic hydrolysis (60 min): HCl4: 2M HCl at 70 °C; HCl5:1M HCl at 100 °C. (n = 3 ± SD).

| **Exp** | **Quercetin**  **(mg/g)** | **Kaempferol**  **(mg/g)** | **Isorhamnetin**  **(mg/g)** | **Myricetin**  **(µg/g)** |
| --- | --- | --- | --- | --- |
| Sna7 | 2.57 ± 0.29^a^ | 1.07 ± 0.12^a^ | 0.29 ± 0.03^a^ | 90 ± 10^a^ |
| Cbi3 | 1.52 ± 0.05^b^ | 0.770 ± 0.022^b^ | 0.1496 ± 0.0026^b^ | 63.7 ± 0.8^b^ |
| Cel1 | 0.344 ± 0.026^d^ | 0.0591 ± 0.005^e^ | 0.0166 ± 0.0018^d^ | n.d. |
| Glu3 | 0.83 ± 0.05^c^ | 0.522 ± 0.025^c^ | 0.146 ± 0.007^b^ | 27.2 ± 1.2^d^ |
| Pec3 | 0.674 ± 0.025^cd^ | 0.306 ± 0.012^d^ | 0.068 ± 0.004^c^ | 20.6 ± 1.1^d^ |
| HCl4 | 1.53 ± 0.11^b^ | 0.68 ± 0.03^b^ | 0.139 ± 0.006^b^ | 63 ± 4^b^ |
| HCl5 | 1.21 ± 0.09^b^ | 0.68 ± 0.04^b^ | 0.123 ± 0.009^b^ | 49 ± 5^c^ |
| Mean values with different letters (a, b, etc.) within the same column are statistically different (*p < 0.05*). Isorhamnetin, kaempferol and myricetin calculated as quercetin equivalents.  n.d. not detected | | | | |

Table S21 Aglycone yields from leaf extracts of R. pseudoacacia. Enzymatic hydrolysis (25 min): Sna7: 5 mg Snailase (pH 5.5, 37 °C); Cbi3: 5 U Cellobiase (pH 4.5, 37 °C); Pec3: 20 U Pectinase (pH 4.5, 40 °C); Glu3: 20 U β-Glucosidase (pH 5.5, 37 °C); Cel1: 20 U Cellulase (pH 5.5, 52 °C). Acidic hydrolysis (60 min): HCl6: 2M HCl at 100 °C. (n = 3 ± SD).

| **Exp.** | **Acacetin**  **(mg/g)** | **Luteolin**  **(mg/g)** | **Quercetin**  **(mg/g)** | **Apigenin**  **(mg/g)** | **Diosmetin**  **(mg/g)** | **Isorhamnetin**  **(µg/g)** |
| --- | --- | --- | --- | --- | --- | --- |
| Sna7 | 2.71 ± 0.05^a^ | 0.307 ± 0.026^a^ | 0.247 ± 0.010^a^ | 0.190 ± 0.012^a^ | 0.136 ± 0.008^a^ | 52 ± 3^a^ |
| Cbi3 | 1.67 ± 0.05^b^ | 0.225 ± 0.014^b^ | n.d. | 0.101 ± 0.005^b^ | 0.076 ± 0.004^b^ | 16.1 ± 0.8^bc^ |
| Cel1 | 0.0236 ± 0.0016^e^ | 0.049 ± 0.005^c^ | 0.0300 ± 0.0026^d^ | 0.0077 ± 0.0008^d^ | 0.0050 ± 0.0004^d^ | n.d. |
| Glu3 | 0.0246 ± 0.0022^e^ | 0.058 ± 0.006^c^ | 0.067 ± 0.006^c^ | 0.0120 ± 0.0009^d^ | 0.0075 ± 0.0008^d^ | 5.2 ± 0.8^d^ |
| Pec3 | 0.958 ± 0.025^c^ | 0.188 ± 0.022^b^ | 0.122 ± 0.013^b^ | 0.084 ± 0.009^b^ | 0.066 ± 0.006^b^ | 20 ± 3^b^ |
| HCl6 | 0.71 ± 0.04^d^ | 0.182 ± 0.012^b^ | 0.108 ± 0.006^b^ | 0.087 ± 0.004^b^ | 0.0477 ± 0.0028^c^ | 11.5 ± 0.6^c^ |
| Mean values with different letters (a, b, etc.) within the same column are statistically different (*p < 0.05*). Acacetin, diosmetin and luteolin calculated as apigenin equivalents; isorhamnetin as quercetin equivalents.  n.d. not detected | | | | | | |

Table S22 Summary of key parameters of the calibration curves.

| **Standard substance** | **Calibration** | **R^2^** | **Range (µg/mL)** | **λ**  **(nm)** | **Equivalents** |
| --- | --- | --- | --- | --- | --- |
| Dihydrokaempferol (DHK) | y = 0.9183x | 0.999 | 0.1-20 | 290 | - |
| Eriodictyol | y = 1.176x | 0.999 | 0.1-50 | 290 | Hesperetin |
| Formononetin | y = 0.6814x | 0.999 | 0.1-20 | 290 | Biochanin A  Genistein |
| Phloretin | y = 0.4741x | 0.998 | 5-500 | 309 | - |
| Apigenin | y = 1.311x | 0.999 | 0.1-20 | 340 | Acacetin  Diosmetin  Luteolin |
| Quercetin | y = 0.5797x | 0.999 | 0.1-50 | 340 | Isorhamnetin  Kaempferol  Myricetin |
| Butein | y = 1.861x | 0.999 | 0.1-100 | 385 | Lanceoletin  Okanin |
| Sulfuretin | y = 2.077x | 0.999 | 0.1-50 | 385 | Leptosidin  Maritimetin |

Table S23 All identified aglycones by UHPLC-ESI-qTOF-MS.

| **Aglycone** | **Sample** | **Molecular formula** | **[M-H]^-^** | **[M-H]^-^_calc._** | **UV**  **(nm)** |
| --- | --- | --- | --- | --- | --- |
| Acacetin | R | C_16_H_12_O_5_ | 283.0615 | 283.0612 | 268, 330 |
| Apigenin | F  Me  R | C_15_H_10_O_5_ | 269.0450  269.0451  269.0458 | 269.0455 | 267, 335 |
| Biochanin A | T | C_16_H_12_O_5_ | 283.0611 | 283.0612 | 260 |
| Butein | B | C_15_H_12_O_5_ | 271.0609 | 271.0612 | 382 |
| DHK | P | C_15_H_12_O_6_ | 287.0567 | 287.0561 | 291 |
| Diosmetin | Me  R | C_16_H_12_O_6_ | 299.0554  299.0560 | 299.0561 | 251, 344 |
| Eriodictyol | Me | C_15_H_12_O_6_ | 287.0563 | 287.0561 | 288 |
| Formononetin | T | C_16_H_12_O_4_ | 267.0662 | 267.0663 | 249, 299 |
| Genistein | T | C_15_H_10_O_5_ | 269.0455 | 269.0455 | 260 |
| Hesperetin | Me | C_16_H_14_O_6_ | 301.0714 | 301.0718 | 287 |
| Isorhamnetin | Ma  Q  R  T | C_16_H_12_O_7_ | 315.0506  315.0504  315.0509  315.0509 | 315.0510 | 254, 370 |
| Kaempferol | F  Ma  P  Q  T | C_15_H_10_O_6_ | 285.0400  285.0406  285.0403  285.0407  285.0409 | 285.0405 | 265, 365 |
| Lanceoletin | B,  C | C_16_H_14_O_6_ | 301.0718  301.0723 | 301.0718 | 382 |
| Leptosidin | C | C_16_H_12_O_6_ | 299.0566 | 299.0561 | 402 |
| Luteolin | B  C  F  Me  R | C_15_H_10_O_6_ | 285.0404  285.0402  285.0402  285.0408  285.0407 | 285.0405 | 253, 346 |
| Maritimetin | B  C | C_15_H_10_O_6_ | 285.0405  285.0405 | 285.0409 | 409 |
| Myricetin | F  Q | C_15_H_10_O_8_ | 317.0304  317.0299 | 317.0303 | 253, 372 |
| Okanin | B  C | C_15_H_12_O_6_ | 287.0563  287.0565 | 287.0561 | 379 |
| Phloretin | Ma | C_15_H_14_O_5_ | 273.0774 | 273.0768 | 287 |
| Quercetin | F  Ma  Q  R  T | C_15_H_10_O_7_ | 301.0353  301.0360  301.0355  301.0353  301.0355 | 301.0354 | 255, 371 |
| Sulfuretin | C | C_15_H_10_O_5_ | 269.0451 | 269.0455 | 392 |
| B: *Bidens ferulifolia*; C: *Coreopsis grandiflora*; F: *Fagus sylvatica*; Ma: *Malus × domestica*; Me: *Mentha × piperita*;  P: *Petunia × hybrida*; Q: *Quercus robur*; R: *Robinia pseudoacacia*, T: *Trifolium pratense* | | | | | |
